# Loss of *Smad4* drives vascular malformations via c-KIT–dependent high–fluid shear stress mimicry

**DOI:** 10.1172/JCI197609

**Published:** 2026-10-01

**Authors:** Johannes Gahn, Fan Wu, Qing Zhang, Yuxi Di, Tanmaya Behera, Yonggang Ren, Zohrah Hashemi, Kuheli Banerjee, Julio Cordero, Claudia Gherman, Kornelia Andorfer, Caroline T. Seebauer, Fatemeh Mirzapour-Shafiyi, Gergana Dobreva, Martin A. Schwartz, Roxana Ola

**Affiliations:** 1 Experimental Pharmacology Mannheim (EPM) and; 2 Department of Cardiovascular Genomics and Epigenomics, European Center for Angioscience (ECAS), Medical Faculty Mannheim, Heidelberg University, Mannheim, Germany.; 3 German Centre for Cardiovascular Research (DZHK), Mannheim, Germany.; 4 Ion Chiricuta Oncological Institute, Cluj Napoca, Romania.; 5 Department of Otorhinolaryngology, Regensburg University Medical Center, Regensburg, Germany.; 6 Department of Otorhinolaryngology, Head and Neck Surgery, Luzerner Kantonsspital, Lucerne, Switzerland.; 7 Center for Synthetic Biology, Technical University Darmstadt, Darmstadt, Germany.; 8 Yale Cardiovascular Research Center, Departments of Internal Medicine (Cardiology), Cell Biology, and Biomedical Engineering, Yale School of Medicine, New Haven, Connecticut, USA.

**Keywords:** Cell biology, Genetics, Vascular biology, Cardiovascular disease, Molecular biology, Signal transduction

## Abstract

Vascular endothelial cells (ECs) encode a homeostatic fluid shear stress (FSS) set point that is essential for vascular stability. Deviations above or below this threshold trigger adaptive remodeling to restore physiological shear levels. Disruption of this control mechanism leads to enlarged arteriovenous malformations (AVMs) in hereditary hemorrhagic telangiectasia, a vascular disorder caused by heterozygous loss-of-function (LOF) mutation in *ALK1*, *ENG*, or *SMAD4*. Mechanistically, *Smad4*-deficient ECs are reset to a lower FSS set point value, resulting in AVMs that show characteristics of high-FSS remodeling with elevated KLF4 and high activation of the downstream Akt. Here, we investigated the KLF4/Akt upstream mechanisms by which SMAD4 sets the physiological FSS set point. We identified the receptor tyrosine kinase c-KIT as a component and regulator of the junctional mechanosensory receptor complex, which is highly upregulated in murine and human AVMs. SMAD4 restrains flow signaling by limiting c-KIT–dependent ERK5 activation and *KLF4* induction. Thus, *SMAD4* LOF leads to sustained c-KIT engagement in the sensory junctional apparatus, driving enhanced and prolonged activation of the ERK5/KLF4/Akt signaling axis. These results show that *Smad4*-LOF mutations induce malformations by disabling a key homeostatic mechanism and identify c-KIT as a potentially previously unrecognized therapeutic target.

## Abstract 2

HHT-like AVMs result from loss of FSS mediated c-KIT repression and inactivation, leading to enhancedmechanosensory complex signaling via SFK hyperactivation driving augmented Erk5-Klf4-pAkt pathway.

## Graphical abstract

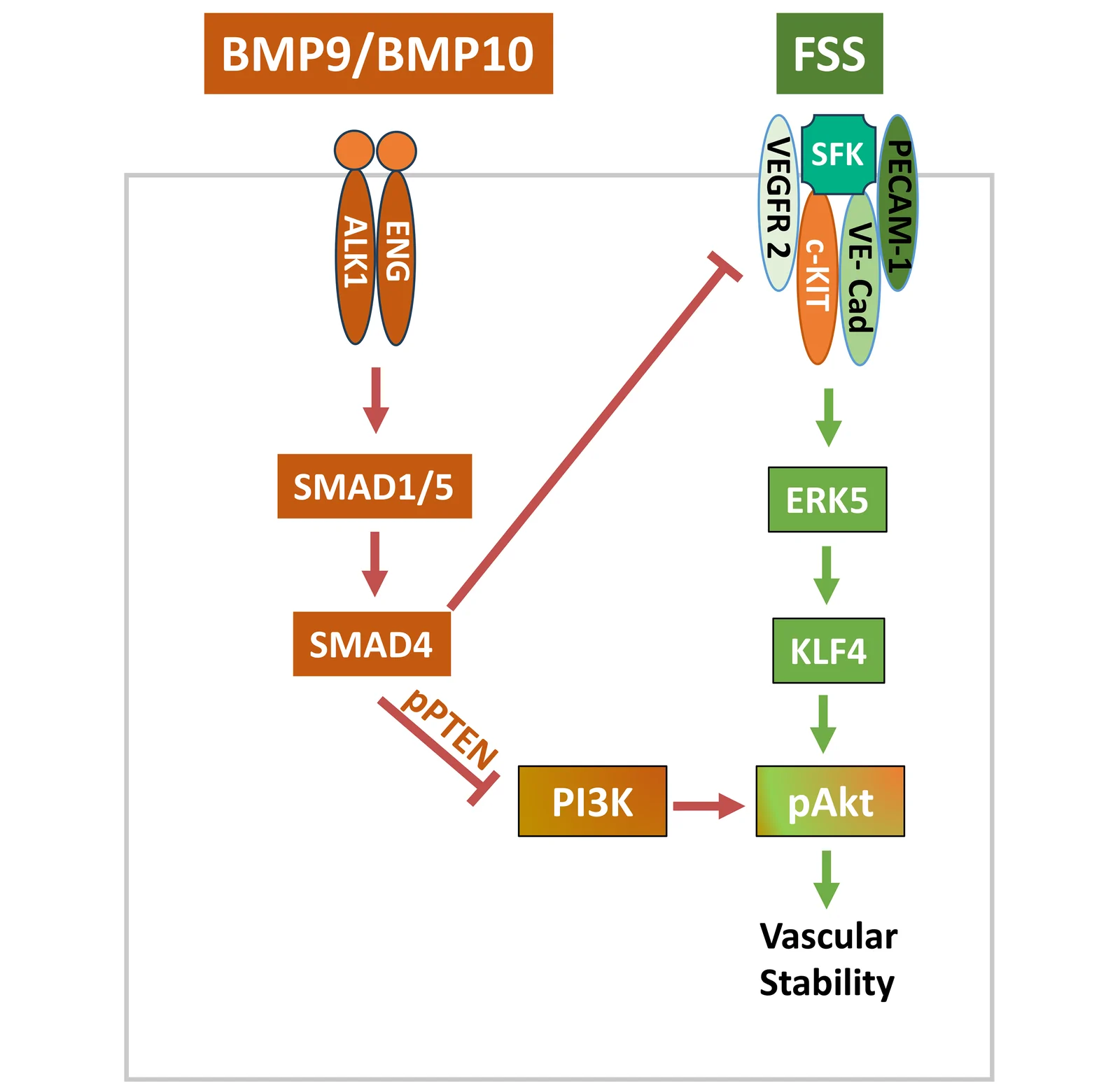

## Introduction

Vascular endothelial cells (ECs) act as professional transducers of fluid shear stress (FSS) from blood flow (1). ECs encode an FSS set point, such that FSS within the physiological range stabilizes vessels in part by limiting inflammatory and remodeling pathways (2). This mechanism presumably prevents remodeling in response to transient changes in blood flow or other stressors. In contrast, sustained FSS below or above this range induces inward or outward vessel remodeling to return FSS toward the physiological set point, at which time stability is restored. Disruption of stabilization pathways is associated with uncontrolled remodeling and vascular disease (3–8).

FSS is sensed in part through a protein complex at cell-cell junctions containing platelet endothelial cell adhesion molecule (PECAM-1), vascular endothelial cadherin (VE-Cadherin), vascular endothelial growth factor receptors 2 and 3 (VEGFR2/3), latrophilin-2 (LPHN2), neuropilin-1, and Plexin D1 (PlxnD1). Flow or direct force application to PECAM-1 or PlxnD1 activates an SRC family kinase (SFK) within seconds. VE-Cadherin functions as an adaptor protein that binds VEGFRs and promotes ligand-independent transactivation of VEGFRs by an SFK (9–14), with data implicating both FYN and YES (15–17). Activated VEGFR2/3 associate with and activate PI3K, which mediates the downstream activation of Akt and integrins (14, 18). Subsequent regulation of NOS3 and NO, RHO GTPases, cytoskeletal organization, and gene expression control EC functions, including vasodilation, vascular remodeling, and susceptibility to atherosclerosis (19, 20). Cell-cell junctions also contribute to activation of ERK5 and induction of transcription factors Krüppel-like transcription factor (*KLF2*) and *KLF4* (21–23), which further maintain physiological Akt activation levels (4). In arteries under physiological flow, KLF2/4 are critical to stability and healthy function, though in other contexts, they can promote growth and remodeling in angiogenesis and in vascular malformations (24–26).

We recently reported complex interactions between activation of SMAD1/5 and induction of *KLF4/2* by FSS. *KLF4* induction by FSS is limited by SMAD1/5/SMAD4 signaling in the low to physiological range, i.e., at or below the set point (4). SMAD1/5 activation by FSS occurs through activin like 1 kinase (ALK1)/ENDOGLIN (ENG) receptors for bone morphogenic factor 9 (BMP9) and BMP10, which appears to be independent of the PECAM-1/VE-Cadherin/VEGFR complex (27). Deletion of *SMAD4*, which blocks SMAD1/5-dependent gene expression, results in enhanced *KLF4* and downstream PI3K/Akt at these FSS magnitudes, which contributes to lesion formation (4). However, under high FSS (above set point), increased *KLF2* suppresses the SMAD1/5 pathway by inducing the BMP/ALK1 inhibitor BMP binding endothelial regulator, to allow outward artery remodeling (6). Together, these results demonstrate that dose-dependent crosstalk between *KLF2/4* and SMAD1/5 sets the set point and stabilizes vessels under physiological FSS while promoting remodeling under higher flow.

Consistent with the critical role of this network in vascular homeostasis, loss-of-function (LOF) mutations in *ALK1/ENG* or *SMAD4* lead to unstable arteriovenous malformations (AVMs) in human hereditary hemorrhagic telangiectasia (HHT) (28–30). HHT lesions originate in nonarterial ECs and are characterized by loss of EC quiescence (31–35), dependent on augmented activation of KLF4/Akt by FSS (4, 36, 37). Thus, *Smad4*-deficient ECs in HHT have a lower FSS set point and exhibit characteristics of high-FSS outward remodeling, which may be interpreted as a futile homeostatic mechanism.

The present study aimed to elucidate the upstream regulatory mechanisms controlling KLF4/Akt signaling and their contributions to AVM pathogenesis. We identified the receptor tyrosine kinase (RTK) c-KIT as a component of the endothelial mechanosensory receptor complex. Our data show that loss of *SMAD4* disrupts physiological flow-dependent regulation of c-KIT, leading to its persistent junctional localization and amplification of the MEKK3/ERK5/KLF4/Akt mechanotransduction cascade. Activation of this axis leads to heightened EC activation, maladaptive chronic remodeling, and AVM formation. These findings thus advance our understanding of EC mechanobiology and uncover c-KIT as a promising therapeutic target for flow-associated vascular diseases.

## Results

### SMAD4 restricts both the magnitude and duration of flow-induced signaling.

We previously reported that SMAD4 restrains flow induction of *KLF4* and activation of Akt to maintain the FSS set point (4). To investigate upstream mechanisms, we assessed flow signaling through the junctional complex in HUVECs subjected to laminar FSS (1-5-12 dynes/cm^2^) for 2 h (h) with/without *SMAD4* using short interfering RNA (siRNA). Specificity of the *SMAD4* siRNA and SMAD4 antibody was confirmed by Western blot (WB) (Figure 1, A and B). We examined FSS-induced phosphorylation of SRC family kinases (SFK^Y416^), VE-Cadherin^Y658^, and VEGFR2^Y1054^; the downstream phosphorylation of Akt^S473^; and the phosphorylation-dependent ERK5 gel shift (upper band) as markers of activation (Figure 1A). When ECs were subjected to FSS at increasing magnitudes, in comparison with control siRNA (*CTRL* siRNA), *SMAD4* knockdown (*SMAD4* KD) augmented FSS-dependent activation of VE-Cadherin, VEGFR2, ERK5, and Akt. Interestingly, *SMAD4* KD not only increased phosphorylation of SFK^Y418^ and VE-Cadherin^Y658^ at low physiological FSS but also prevented their inhibition at high FSS (Figure 1A, quantified in Figure 1B). Thus, SMAD4 restricts mechanotransduction pathways upstream of KLF4/Akt to establish the FSS set point.

We next assayed effects of *SMAD4* loss on these pathways over 5–240 minutes under 12 dynes/cm^2^ FSS, a typical physiological level (Figure 1, C–E). SFK activation is the earliest flow-responsive event downstream of PECAM-1, followed by phosphorylation of VE-Cadherin, VEGFRs, PI3K, and Akt (14). *SMAD4* depletion increased SFK^Y416^, VE-Cadherin^Y658^, and Akt^S473^ in this context as well and converted the transient activation to a sustained time course, suggesting loss of a physiological negative feedback mechanism. FSS-mediated ERK5 activation was also markedly amplified, driving excessive *KLF4* transcription and persistent Akt activation (Figure 1, C–E). Sustained junctional component activation and heightened SFK/ERK5/KLF4 signaling after *SMAD4* KD were also evident at 24 h (Supplemental Figure 1, A–E; supplemental material available online with this article; https://doi.org/10.1172/JCI197609DS1). Taken together, these data suggest that SMAD4 restricts FSS signals through the junctional mechanosensory receptor complex in a time- and magnitude-dependent manner.

### SMAD4 restricts junctional mechanotransduction upstream of MEKK3/ERK5/KLF4/Akt activation.

We next evaluated the role of sustained SFK activity in downstream ERK5/KLF4/Akt signaling. *CTRL* and *SMAD4* siRNA ECs were examined at 2 h under 12 dynes/cm^2^ FSS with and without the SFK inhibitor, PP2. SFK inhibition reduced ERK5 and the downstream *KLF4*/Akt activation in both *CTRL* and *SMAD4* siRNA cells (Figure 2, A–C). As SFKs are essential for activation of the mechanosensory receptor complex, we next addressed the requirement for junctional PECAM-1, VE-Cadherin, and VEGFRs. Depletion of any of these components reduced the high ERK5/*KLF4*/Akt levels in *SMAD4* siRNA HUVECs (Figure 2, D–F, and Supplemental Figure 2, A and B). Together, these results support a requirement for junctional SFK activation within the mechanosensory receptor complex for augmented ERK5/*KLF4*/Akt after *SMAD4* KD.

To determine the functional role of ERK5 in the context of *SMAD4* LOF, HUVECs were additionally depleted of *MEKK3* (Figure 2, G–I) or treated with the MEK5 inhibitor, BIX02189 (Supplemental Figure 2, D–F). Cells were subjected to 12 dynes/cm^2^ FSS for 2 h and signaling pathways were assayed. *SMAD4* KD induced the MEKK3 gel shift, indicating its activation (Figure 2G and Supplemental Figure 2C). Inhibition of MEK5-MEKK3 abrogated FSS-mediated ERK5 activation and *KLF4* induction, and the downstream Akt activation, in both control and *SMAD4* KDECs (Figure 2, G–I, and Supplemental Figure 2, D–F).

To assess ERK5 activation in vivo, we labeled postnatal day 6 (P6) retinas from *Smad4^fl/fl^* and *Smad4*^iΔEC^ (EC-specific tamoxifen-inducible [Tx-inducible] *Smad4* deletion) neonates for p-ERK5 (green), KLF4 (green), phosphorylated ribosomal S6 (p-S6, green), along with Isolectin B4 (IB4; white) to identify ECs (Figure 2J). In control *Smad4^fl/fl^* retinas, ERK5 activation was stronger in arteries than in veins. In *Smad4*^iΔEC^ retinas, increased ERK5 marked the entire vascular plexus, including the AVMs. KLF4 was highly elevated; and ribosomal S6, which is downstream of Akt, was highly phosphorylated (Figure 2, J and K) (4, 36). Thus, derepression of MEKK3-ERK5 in *Smad4* KO/KD ECs augments KLF4 expression and Akt activation in AVM lesions.

### Upregulation of c-KIT in high-flow but low-FSS AVMs.

We assessed FSS levels in retinal AVMs using computational flow simulation on the PolNet platform (38). As previously reported in murine wild-type developing retinas, the simulated wall shear stress (WSS) is the lowest at the sprouting front, is low to medium in the veins, and the highest in arteries and first branch point capillaries, with the latter being the region prone to AVM formation (27, 35). Interestingly, simulated WSS within the AVM in *Smad4*^iΔEC^ was lower (between 1 and 5 Pascals) compared with *Smad4^fl/fl^* retinal arteries and veins. In vessels beyond the AVMs (toward periphery), WSS was nearly 0 (Figure 3, A and B), accounting for low KLF4 in these regions (39). Although flow rates within AVMs are relatively high, FSS is inversely proportional to vessel radius (40). As a result, ECs within these enlarged, high-flow AVMs experience low FSS while exhibiting activation of pathways typical of high FSS.

To investigate the mechanisms upstream of SFK/ERK5/KLF4/Akt driving sustained mechanotransduction upon *SMAD4* loss, we performed bulk RNA sequencing (RNA-seq) in HUVECs with/without *SMAD4*, grown statically or under low, medium, and physiological FSS (1-5-12 dynes/cm^2^) for 2 h (Figure 3C). Principal component analysis revealed a clear separation between the *CTRL* and *SMAD4* siRNA groups (Supplemental Figure 3A). Major gene ontology (GO) terms for the differentially expressed genes (DEGs) upregulated upon *SMAD4* KD (comparison of all *CTRL* versus *SMAD4* siRNA groups) included MAPK pathway, cell migration, vascular development, and cell adhesion, all of which are pertinent to EC mechanobiology (Figure 3D). Among the downregulated DEGs, the cell cycle–related terms predominated (Supplemental Figure 3, D and E). Interestingly, listing the top 5 upregulated genes within each GO term, *KIT* gene was present in 5 out of the 8 GO terms (Figure 3D). Reactome pathway analysis further identified upregulated c-KIT signaling, thus suggesting its role in FSS pathway deregulation after *SMAD4* LOF (Supplemental Figure 3B). We therefore focused on the c-KIT RTK, the receptor for stem cell factor (SCF), encoded by *KITL*.

Visualizing RNA-seq samples in Ridgeplot, *KIT* in *CTRL* siRNA cells was downregulated by FSS at all magnitudes. In *SMAD4* KD cells, *KIT* was strongly upregulated under static conditions, and the downregulation by flow was reduced, resulting in higher levels of *KIT* under all conditions (Supplemental Figure 3C). Independent analysis of mRNA by RT-PCR revealed similar strong upregulation by *SMAD4* siRNA without flow and minimal downregulation by flow in *SMAD4* KD cells, with a decreasing trend for *KIT* mRNA from lower to higher flow (Figure 3E). Protein analysis showed that *SMAD4* KD upregulated c-KIT protein and blocked its inhibition by flow of all magnitudes (Figure 3, F and G). *SMAD4* loss thus potently upregulates *KIT* via both transcriptional and posttranscriptional mechanisms.

We next assessed BMP9 in regulation of *KIT* by FSS. HUVECs under static versus physiological FSS were stimulated with 1 ng BMP9 for 2 h. Similar to FSS, BMP9 downregulated *KIT* expression, which required SMAD4 (Figure 3H) and augmented the inhibitory effect of FSS on *KIT* expression (Figure 3I). To investigate whether c-KIT is similarly regulated by SMAD4 upstream signaling components, HUVECs were depleted for *SMAD1/5* and *ALK1* and stimulated with 12 dynes/cm^2^ FSS for 2 h. Similar to *SMAD4* depletion, loss of either *ALK1* or *SMAD1/5* led to an increase in *KIT* expression in static and in flow conditions but also to augmented activation of ERK5/*KLF4*/Akt upon physiological FSS (Supplemental Figure 3, F–I). Thus, c-KIT and heightened flow signaling are part of a conserved mechanism across multiple AVM models.

### SMAD4 restricts c-KIT expression in vivo.

To corroborate our findings in vivo, we first reanalyzed published single-cell RNA-sequencing (scRNA-seq) data from P6 wild-type retinas (41). *Kit* transcripts were expressed at low levels in tip cells and some venous like ECs and absent in arterial ECs (Figure 4A). Labeling P6 *Smad4^fl/fl^* versus *Smad4*^i*Δ*EC^ retinas for c-KIT showed enhanced expression of c-KIT in tip cells and a few capillaries of *Smad4^fl/fl^* (arrowheads in Figure 4B, quantified in Figure 4, C and D), consistent with scRNA-seq results and confirming antibody specificity. In *Smad4*^iΔEC^ retinas, c-KIT was strongly upregulated in all ECs, including AVMs (arrowheads in Figure 4B, quantified in Figure 4, C and D).

We next examined c-KIT expression in 2 specimens from human HHT type 2 (HHT2) nasal telangiectasia biopsies and compared AVM region versus non-AVM regions within the same biopsy. Sections were stained for c-KIT, ENDOGLIN (to visualize the endothelium), α–smooth muscle actin (α-SMA; for smooth muscle cells), and DAPI (to visualize the nuclei). We observed a segment of the AVM endothelium strongly positive for c-KIT (blue versus yellow arrowheads in Figure 4E, quantified in Figure 4F), suggesting de novo expression, while the arterial and venous endothelium within the non-AVM regions was negative. Notably, mast cells also stained strongly for c-KIT. Thus, *Smad4* inactivation triggers c-KIT upregulation in both mouse and human lesion ECs in vivo.

### SMAD4 loss promotes c-KIT–dependent amplification of junctional flow signaling.

We next considered the functional role of c-KIT in FSS mechanotransduction. Specificity of *KIT* siRNA and c-KIT antibody was confirmed by immunoblotting (Supplemental Figure 4A). In control cells, *KIT* KD had very little effect on FSS-mediated mechanotransduction (Supplemental Figure 4, A–C). Yet, in the context of *SMAD4* depletion, *KIT* KD reduced the elevated p-SFK and p–VE-Cadherin and ERK5/*KLF4*/Akt activation, all components of or downstream of the junctional receptor complex (Figure 5, A–C). Thus, upregulated c-KIT in *SMAD4* KDECs leads to increased activation of junction-dependent signals.

To determine whether c-KIT acts within the mechanosensory receptor complex, we immunoprecipitated c-KIT and VE-Cadherin and probed immunoprecipitants for junctional components using cells with/without *SMAD4* under static and 12 dynes/cm^2^ FSS conditions (Figure 5D and Supplemental Figure 4, D and E). c-KIT associated with VE-Cadherin and VEGFR2 under static and flow, in both control and *SMAD4* KD cells (Figure 5D). VE-Cadherin immunoprecipitation (IP) confirmed interaction with c-KIT and VEGFR2 (Figure 5E). Upon 2-hr FSS, control ECs showed reduced c-KIT expression and association with VE-Cadherin, despite comparable VE-Cadherin IP efficiency, accompanied by diminished VE-Cadherin Tyr phosphorylation. In contrast, *SMAD4*-deficient cells retained elevated c-KIT expression and sustained junctional association with increased VE-Cadherin activation, consistent with prolonged c-KIT–dependent junctional signaling (Figure 5, D and E). c-KIT thus shows physical and functional interactions with the junctional complex after its upregulation by *SMAD4* LOF.

### KIT inhibition in SMAD4 KD cells restores the FSS set point for EC behaviors.

We next evaluated the role of c-KIT in the enhanced FSS-mediated EC behaviors in *SMAD4* KD cells. We first assessed the EC elongation (ratio of length to width of individual ECs) and the EC alignment parallel to the direction of flow (≤30° angle directionality) in HUVECs depleted of *KIT* or *SMAD4*, or with double depletion of *KIT* and *SMAD4*, grown in static versus 1 and 12 dynes/cm^2^ FSS for 48 h (Figure 6, A–D). Under both magnitudes, HUVECs depleted of *KIT* alone showed no difference compared to *CTRL* cells. By contrast, *KIT* KD in *SMAD4* KD cells restored the augmented flow-induced EC elongation and alignment (Figure 6, A–D). We next labeled cells for the Golgi apparatus (GM130, red), VE-Cadherin (white), and DAPI (blue), and we measured the nucleus-Golgi axis angle (at an angle ≤ 45° in relation to EC orientation) (Supplemental Figure 4, F and G; quantified in Figure 6, E and F). As previously shown (4), *SMAD4* KD HUVECs oriented against the flow direction under 1 dyne, whereas *KIT*-KD cells showed no orientation (Supplemental Figure 4F and Figure 6E). Under 12 dynes/cm^2^, KD of *SMAD4* reduced orientation, whereas *KIT* KD similar to *CTRL* cells oriented against the flow direction (Supplemental Figure 4F; quantified in Figure 6F). Interestingly, *KIT* KD restored the aberrant axial polarity in *SMAD4*-depleted HUVECs under both low and physiological FSS ((Supplemental Figure 4, F, and G; quantified in Figure 6, E and F).

To further assess the effect of *KIT* KD in EC proliferation, we performed 5-ethynyl-2′-deoxyuridine (EdU) labeling to identify ECs in S phase. In both *KIT* and *CTRL* siRNA cells, 12 dynes/cm^2^ promoted cell cycle arrest, while 1 dyne had no effect (Figure 6, G and H). In contrast, 1 dyne/cm^2^ reduced DNA synthesis in *SMAD4*-depleted HUVECs, which was reversed by *KIT* siRNA (Figure 6G). Together, these results show that resetting the FSS set point to lower values by *SMAD4* KD requires c-KIT.

### Soluble ligand–independent c-KIT activation in SMAD4 KD sustains flow signaling.

We next investigated whether c-KIT function in mechanotransduction requires its ligand, SCF. In our RNA-seq dataset, *KITL* was selectively upregulated under physiological FSS only in *SMAD4* KD cells (Figure 7A), which was confirmed by qPCR (Figure 7B). To elucidate the role of SCF in mediating c-KIT events, we blocked SCF using a specific neutralizing antibody (SCF blAb). Interestingly, SCF blockade did not attenuate the elevated SFK/ERK5/*KLF4*/Akt activation in *SMAD4* KD cells (Figure 7, C–E). Conversely, stimulation with the soluble SCF transiently increased p-SFK and p-Akt levels at 5 minutes in both *CTRL* and *SMAD4* siRNA cells, but it did not further enhance ERK5/*KLF4* activation (Figure 7, F–H). Collectively, these data suggest that c-KIT driving augmented and persistent mechanotransduction in *SMAD4* KD cells occurs independently of its soluble ligand.

We next tested the effects of avapritinib, an FDA-approved selective inhibitor of c-KIT catalytic activity, which also targets PDGFRA (42, 43). Avapritinib efficiently suppressed SCF-induced c-KIT phosphorylation at Y719 (PI3K activation site) (44) and downstream SFK, Akt, and ERK1/2 activation in *CTRL* and *SMAD4* KD cells, confirming pathway suppression (Supplemental Figure 5, A and B).

Interestingly, in HUVECs with/without *SMAD4* subjected to 2 h of 12 dynes/cm^2^ FSS, pharmacological inhibition of c-KIT activity selectively rescued FSS-induced junctional SFK and the ERK5/*KLF4*/Akt activation (Figure 7, I–K). Analysis of our RNA-seq data showed that *PDGFRA* was unchanged following *SMAD4* depletion, despite robust induction of *KIT*, suggesting that altered PDGFRA signaling is unlikely to account for the observed effects of avapritinib.

### Inhibition of c-KIT kinase activity reverses AVM formation.

We next assessed the effect of avapritinib in rescuing AVM formation in *Smad4*^iΔEC^ mice. Tx was administered at P0 and P1 in *Smad4^fl/fl^* and *Smad4*^iΔEC^ littermates, followed by intraperitoneal (i.p.) administration of avapritinib at P3–P5, with retinas analyzed at P6 (Figure 8A). Avapritinib rescued the number of AVMs and the increased vascular density at the angiogenic front in *Smad4*^iΔEC^ retinas, whereas no effect was observed in *Smad4^fl/fl^* littermates (Figure 8, B–E).

To analyze the role of c-KIT activation in cell cycle progression, we labeled retinas of *Smad4^fl/fl^* and *Smad4*^iΔEC^ DMSO- and avapritinib-treated neonates for IB4 (red), ERG (green), and KI67 (white). Avapritinib treatment reduced the high EC proliferation in the *Smad4*-deficient vascular plexus with no effect on *Smad4^fl/fl^* (Figure 8, D and F). We further evaluated axial polarity by labeling retinas for ERG1 (white), GOLPH4 (red), and IB4 (green) (Figure 8G). Compared with DMSO-treated *Smad4*^iΔEC^, avapritinib treatment blunted the increased axial polarity in *Smad4*^iΔEC^ (Figure 8, G and H). Thus, elevated c-KIT kinase activity promotes cell cycle progression and aberrant EC migration in *Smad4*^iΔEC^ leading to AVM formation.

Last, we tested avapritinib in established AVMs by administering the inhibitor at P4 (Supplemental Figure 5, C–F). In this setting, avapritinib did not change the number of AVMs but significantly decreased their diameter (Figure 8, E and F). To validate rescue of AVMs by c-KIT inhibition, *Smad4*^iΔEC^ neonates in an mTmG background (that allows visualization of gene recombination upon Tx administration) were treated with masitinib, another potent and selective RTK inhibitor that was shown to target c-KIT (45). Masitinib also rescued the number of AVMs in *Smad4*^iΔEC^ mice (Supplemental Figure 5, G and I). Thus, 2 distinct inhibitors of c-KIT kinase activity are potential treatments for AVMs in patients with HHT.

## Discussion

ECs sense biomechanical cues that help maintain vascular stability. We previously reported that loss of SMAD4 signaling downstream of canonical BMP9 and BMP10 shifts EC responses to a lower FSS set point (4). In unperturbed ECs, this pathway is maximally activated at physiological FSS, where it induces genes that promote quiescence and vascular stability, with suppression at higher FSS (6). *Smad4* KO/KD ECs show elevated FSS-induced KLF4 and Akt activation linked to high-FSS outward remodeling. These results suggest that ECs within AVMs misread flow as nonphysiological and chronically activate remodeling in a futile attempt to restore FSS to the correct range, resulting in fragile, dilated, rupture-prone vascular malformations. Consistent with this model, computational fluid dynamics shows that WSS in high-flow retinal AVMs is below normal due to the larger vessel diameter. Thus, HHT lesions involve conversion of the homeostatic remodeling response to a chronic, self-perpetuating remodeling state associated with reduced vessel stability.

Overactivated KLF4 in AVMs induces TIE2 expression, which results in augmented Akt activation (4, 36). This mechanism synergizes with reduced PTEN activity, PIP3 accumulation, and a further activation of PI3K/Akt and other downstream pathways in lesion ECs (32, 34, 35, 46, 47). Here, we aimed to identify the mechanism by which SMAD4 restricts FSS-mediated KLF4/Akt activation and to relate these events to AVM pathophysiology.

ECs respond to onset of FSS through transient signaling responses that typically return to baseline over time. Downstream of LPHN2, PlxinD1, or PECAM-1, SFK activation leads to phosphorylation of VE-Cadherin and VEGFRs and subsequent activation of PI3K/Akt (14). These early junctional mechanosensing events set in motion signaling cascades that lead to long-term changes in EC gene expression and cell shape and cytoskeletal organization by which cells adapt to the flow environment. ERK5, downstream of MEKK2/3-MEK5, is activated within 30 minutes and remains high through a dual regulatory mechanism involving sustained MEKK2/3 signaling (22) and mitochondria-dependent activation (48). Nuclear ERK5 thereafter induces the expression of *KLF2/4*, which at physiological levels contribute to vessel stability (22). Yet, how the magnitude and duration of these signals are dynamically regulated remains poorly understood.

Here, we show that *SMAD4* loss leads to enhanced and sustained activation of junctional events, including SFK, VE-Cadherin, and the downstream ERK5/KLF4/Akt axis, converting and amplifying a transient flow response into a prolonged, elevated signaling state. Surprisingly, we identified the RTK c-KIT as a key mediator of this dysregulation. *SMAD4* loss prevents the FSS-mediated repression of c-KIT, resulting in its sustained accumulation within the junctional complex. In line with its nonessential role in physiological vascular development (49), c-KIT is also dispensable for physiological flow signaling. Yet, depletion of *KIT* restored physiological FSS signaling and EC behaviors in *SMAD4*-deficient cells, defining c-KIT as a context-dependent amplifier of pathological flow responses, consistent with its reported role in pathological angiogenesis in ocular diseases (50) and cancer (51).

Interestingly, the mechanisms linking FSS to activation of MEKK2/3 in normal ECs remain largely unclear. Plating cells in low confluency or blockade of junctional LPHN2 and PlxinD1 attenuates FSS induction of *KLF2/4* by 50%–70% (unpublished work from MAS’s laboratory) (12, 13), whereas single depletion of PECAM-1/VEGFR2/VE-Cadherin has minimal effect (4), suggesting redundancy of mechanosensory inputs. In contrast, under *SMAD4*-deficient conditions, inactivation of PECAM-1 or VEGFR2/3, and to a lesser extent VE-Cadherin, suppresses *KLF4* activation. These findings indicate that *SMAD4* loss shifts the system from a redundant and distributed signaling architecture to a more junction-dependent state, likely reinforced by c-KIT–mediated amplification along with increased junctional PECAM-1 enrichment (4). As a result, flow signaling becomes exaggerated, promoting sustained EC activation and pathological remodeling. Aberrant ERK5/KLF2/4 activation is also an important driver of disease in cerebral cavernous malformation (CCM) ECs (52). These findings thus point toward shared features between CCM and HHT vascular malformations.

Similar to flow-dependent activation of other RTKs, e.g., VEGFRs and TIE2 (14, 53), c-KIT activation occurs independently of its soluble ligand. For VEGFR2, flow activation is through SFK-dependent phosphorylation and transactivation. These results suggest that under *SMAD4* deficiency, upregulated c-KIT localizes to cell-cell junctions and is transactivated by a similar mechanism. These findings extend the role of c-KIT beyond its canonical function as a ligand-activated receptor and position it as a regulator of endothelial mechanobiology.

c-KIT and ERK5/KLF4/Akt are highly expressed or activated in ECs within murine and human AVMs, supporting in vivo pathological relevance. Inhibiting c-KIT kinase activity attenuates Smad4 loss–associated EC proliferation, and shifts axial polarity in *Smad4*-deficient vessels, decreasing the number of AVMs if given early and reducing the size of established AVMs if given later. These findings identify c-KIT as a druggable target for treating patients with HHT.

This study has several limitations. Most important is the lack of reliable phosphorylated c-KIT antibodies, which prevented direct assessment of c-KIT activation states. Second, potential off-target and non-cell-autonomous effects of c-KIT kinase inhibitors cannot be fully excluded; future studies using genetic LOF models of *Kit* will be required to validate these findings. In addition, our analyses were focused on neonatal retinal AVMs and 2 HHT2 mucosal biopsies; future studies should extend these findings to additional vascular beds and larger human cohorts including distinct HHT genotypes.

Major yet-unanswered questions in this study include: what are the physiological mechanisms by which Smad4 downregulates c-KIT? how does flow drive ligand-independent activation of c-KIT? does c-KIT coordinate early scaffold-dependent and late kinase-driven mechanotransduction? which specific SFKs mediate these processes? is there an RTK that fulfills a similar role in ERK5/KLF2/4 induction in normal vasculature? will c-KIT kinase inhibitors be safe and effective treatments for patients with HHT?

## Methods

### Sex as a biological variable.

Sex was not considered as a biological variable in the present study. Our study examined male and female neonates, and similar findings are reported for both sexes.

### RNA-seq and analysis.

Total RNA from HUVECs transfected with *CTRL* and *SMAD4* siRNAs and grown in static versus 1-5-12 dynes/cm^2^ conditions for 2 h in 2% FBS was isolated using the Quick-RNA Miniprep Kit (Zymo Research). The RNA 6000 Nano Kit (Agilent) was used to assess the RNA integrity on a Bioanalyzer 2100 (Agilent). Library preparation and sequencing were performed on the BGISEQ-500 platform. Quality of RNA-seq reads was assessed with the MultiQC tool (v1.13) and trimmed of adapters using Trimmomatic (v0.39). Reads were mapped by STAR (v2.7.10a) with the following settings: -alignIntronMin 20 and -alignIntronMax 500,000 to the hg38 reference genome. Tag directories were created with makeTagDirectory, and reads were counted by the analyzeRepeats.pl function (rna hg38 -strad both -count exons -noadj), both from HOMER (v4.7.2). Differential expression was quantified and normalized using DESeq2. Rpkm.default from EdgeR was used to determine average reads per millions mapped (RPKM). Heatmaps were created by using heatmapper.ca from the RPKM values and represent the row-based *Z*-scores.

### Animal experiments.

To generate *Smad4*^iΔEC^ neonates, we intercrossed 8-week-old *Smad4^fl/fl^* mice (*Smad4^tm2.1Cxd^*; The Jackson Laboratory) and Tx-inducible *Cdh5-Cre^ERT2^* mice. To generate mTmG *Smad4*^iΔEC^ mice, we subsequently intercrossed *Smad4*^iΔEC^ with an *mTmG* reporter mouse line (The Jackson Laboratory). Gene deletion was achieved by i.p. injections of 100 μg Tx (Sigma-Aldrich) into *Smad4*^iΔEC^and mTmG *Smad4*^iΔEC^ at P0–P2. Tx-injected Cre-negative littermates (*Smad4^fl/fl^*) were used as controls. c-KIT kinase inhibitor avapritinib (MedChemExpress, 50 mg/kg/d) was administered i.p. either at P3–P5 or at P4–P5, and masitinib (MedChemExpress, 50 mg/kg/d) was administered i.p. at P3–P5. Mice were kept in a controlled environment, adhering to standard specific pathogen–free conditions.

### Quantitative real-time PCR.

RNA was extracted using RNeasy Mini Kit (QIAGEN). The RNA was reverse-transcribed with High-Capacity cDNA Reverse Transcription Kit (Thermo Fisher Scientific), and qPCR was performed using PowerUP SYBR Green Master Mix (Thermo Fisher Scientific) with a QuantStudio 3 (Thermo Fisher Scientific) according to the manufacturer’s protocol. For a detailed list of primer sequences, see Supplemental Table 1.

### Immunostaining.

Eyes of P6 pups or plated cells on coverslips were fixed in 4% paraformaldehyde for 17 minutes at room temperature. Staining with specific antibodies (Supplemental Table 2) was performed as previously described (4). High-resolution images were captured using a Zeiss confocal microscope equipped with an Airyscan Detector and analyzed with Zeiss ZEN software. Quantification of retinal vasculature and of labeled cells was performed using Fiji.

### Cells and cell culture reagents.

HUVECs were obtained from PromoCell (no. 12203) and cultured in Endothelial Cell Growth Medium MV2 (PromoCell, C-22022). Depletion of *SMAD4*, *MEKK3*, *KIT*, *ALK1*, *SMAD1/5*, *KIT*, *CD31*, *CDH5*, *KDR*, and *FLT4* was achieved by transfecting 15 pmol of siRNA using Lipofectamine RNAiMax (Invitrogen) in 2% OptiMEM (Gibco). The sequences of specific siRNAs can be found in Supplemental Table 3. Transfection efficiency was assessed by WB and qPCR. Experiments were performed 48–72 h after transfection, and results were compared with *CTRL* siRNA. Inhibition of MEK5 was achieved by using 10 μM BIX02189, added 2 h before the onset of FSS; 5 μM PP2 was added 30 minutes before FSS; and 1 μM avapritinib was added 10 h before the FSS onset. Corresponding volumes of DMSO were added to *CTRL* cells. SCF pathway stimulation was achieved with 50 ng/mL SCF for 5 and 120 minutes in addition to 12 dynes/cm^2^ flow stimulation. Blockade of SCF using a human-specific blocking antibody (2 μg/mL) was done 1 hr before onset of flow. Before experiments, cells were starved for 4–8 h in 2% FCS. Chemicals are listed in Supplemental Table 4. For long-term flow experiments (48 h), cells were maintained in full medium.

### Exposure of ECs to shear stress.

Following siRNA transfection of HUVECs, fully confluent cells plated in 6-well plates were supplemented with 2 mL of cell culture media. The plates were subsequently placed on an orbital shaker (Rotamax120, Heidolph Instruments), which was set to specific rotations per minute (rpm) to generate shear stress levels of 1, 5, and 12 dynes/cm^2^. The rpm to achieve the desired shear stress was calculated using the following formula (Equation 1):
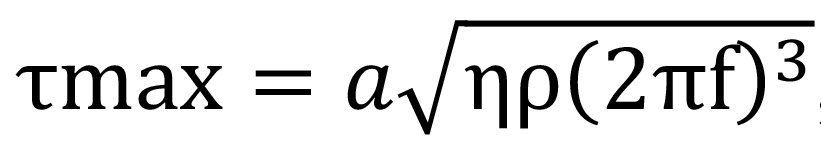


where *a* denotes the orbital radius of rotation in cm, η indicates the viscosity of the medium, ρ represents the density of the culture medium, and f is the frequency of rotation (in rotations per second) (54).

### WB.

HUVECs were washed with PBS and subsequently lysed using Laemmli buffer (Bio-Rad). The resulting samples were separated through 8% or 10% SDS-PAGE gels and transferred onto 0.2 μm nitrocellulose membranes (GE Healthcare, now Cytiva). Western blots were developed with Clarity Western ECL Substrate (Bio-Rad) utilizing a Luminescent Image Analyzer, Fusion FX (Vilber). Band intensities were quantified using Fiji software. The list for primary and secondary antibodies can be found in Supplemental Tables 5 and 6.

### IP.

For the IP procedure, cell lysates were prepared utilizing RIPA buffer (Carl Roth 23T1.3) and subsequently subjected to centrifugation at 17,000*g* for 20 minutes. The supernatants were incubated overnight at 4°C with specific antibodies: c-KIT (Cell Signaling Technology 3074S, 1:50, D13A2), VE-Cadherin (Cell Signaling Technology 2500S, 1:50, D87F2), or Rabbit IgG (Cell Signaling Technology 2729S, 1:200, polyclonal). Following this incubation, protein A/G–coated Dynabeads (Thermo Fisher Scientific, 88803) were incorporated, and the mixture was allowed to incubate for an additional 2 h at 4°C. The beads were washed thoroughly 4 times with PBS containing 0.05% Tween 20. The captured proteins were eluted at 25°C with gentle agitation using a soft elution buffer composed of 50 mM Tris-HCl (pH 8.0), 2% SDS, and 0.1% Tween 20. The resulting eluate was combined in a 1:4 ratio with 4× Laemmli Buffer (Bio-Rad 1610747) and incubated at 65°C for 10 minutes, followed by SDS-PAGE and subsequent immunoblotting analysis.

### Biopsies.

Nasal mucosa biopsies containing AVMs and surrounding non-AVM tissue were subsequently embedded in paraffin and stained as previously described (36). Antibodies used in this study are included in Supplemental Table 2. Slides were imaged with the LSM800 Confocal microscope with Airyscan Detector and the Zeiss ZEN software. Quantification of retina and human samples was done using Fiji.

### Statistics.

Data are expressed as the mean ± SEM. The statistical analysis was performed using the 2-tailed Student’s *t* test as well as 1-way and 2-way ANOVA, followed by Tukey’s method for multiple comparisons, employing GraphPad Prism 9.0 (GraphPad Software). A *P* value of less than 0.05 was considered the lowest statistically significant value.

### Study approval.

The animal procedures received approval from the animal welfare commission of the Regierungspräsidium Karlsruhe, Karlsruhe, Germany. Nasal biopsies containing AVMs and normal surrounding tissue was obtained from patients who underwent surgery for vascular malformations associated with HHT between 2018 and 2023 within the Department of Otorhinolaryngology, University Medical Center Regensburg, Germany, in accordance with the principles of the Declaration of Helsinki and was approved by the local ethics committee, Regensburg, Germany (No. 17-854-101). Written informed consent was obtained from all participants.

### Data availability.

RNA-seq datasets have been deposited at National Center for Biotechnology Information Gene Expression Omnibus with accession number GSE330070. The numerical data underlying all graphs and summary statistics are provided in the Supporting Data Values file.

## Author contributions

JG, FW, and QZ conducted experiments and acquired and analyzed data. The order within the co–first authorship was chosen according to the scientific contributions throughout the manuscript. YD, TB, YR, ZH, KB, JC, CG, KA, CTS, and FMS contributed to the investigation. RO conceptualized and supervised the project and wrote the manuscript. GD and MAS reviewed and edited the manuscript.

## Conflict of interest

The authors have declared that no conflict of interest exists.

## Funding support

This work is the result of NIH funding, in whole or in part, and is subject to the NIH Public Access Policy. Through acceptance of this federal funding, the NIH has been given a right to make the work publicly available in PubMed Central.

- NIH R01: HL169510 — RO, MAS.
- Deutsche Forschungsgemeinschaft: 394046768 - SFB1366 — RO, GD.
- China Scholarship Council: 202307060006 — FW.
- China Scholarship Council: 202308320067 — YD.

## Supplementary Material

Supplemental data

Unedited blot and gel images

Supporting data values

## Figures and Tables

**Figure 1:**
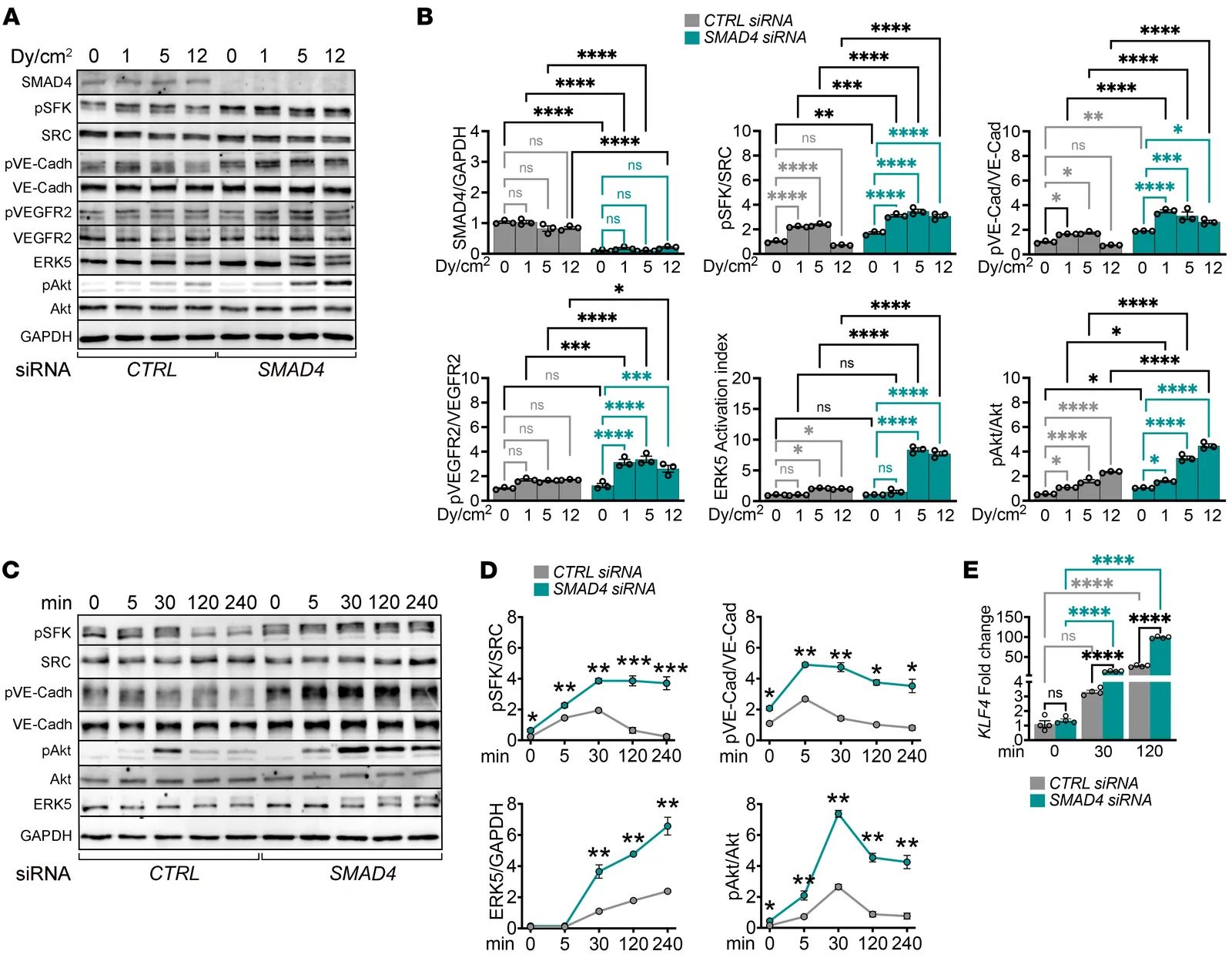
SMAD4 restricts both the magnitude and duration of flow-induced signaling. (**A** and **C**) Western blot (WB) for the indicated proteins in HUVECs transfected with *CTRL* or *SMAD4* siRNAs grown statically or subjected to 1-5-12 dynes/cm^2^ for 2 h (**A**) or subjected to 12 dynes/cm^2^ in a time course of 0–240 minutes (**C**). (**B** and **D**) Quantifications of SMAD4, SFK (Y416), VE-Cadherin (Y658), VEGFR2 (Y1054), and Akt (S473) levels normalized to GAPDH or total SRC, VE-Cadherin, VEGFR2, and Akt, respectively, and of ERK5 gel shift (quantified by densitometry) (*n* = 3/group). p-, phosphorylated. (**E**) qPCR for *KLF4* (fold-change) in *CTRL* and *SMAD4* siRNA HUVECs grown statically (0 minute) versus 30 and 120 minutes of 12 dynes/cm^2^ (*n* = 4). Data are represented as mean ± SEM. Two-way ANOVA was used to determine statistical significance in **B**, **D**, and **E**. *P < 0.05, **P < 0.01, ***P < 0.005, ****P < 0.001.

**Figure 2:**
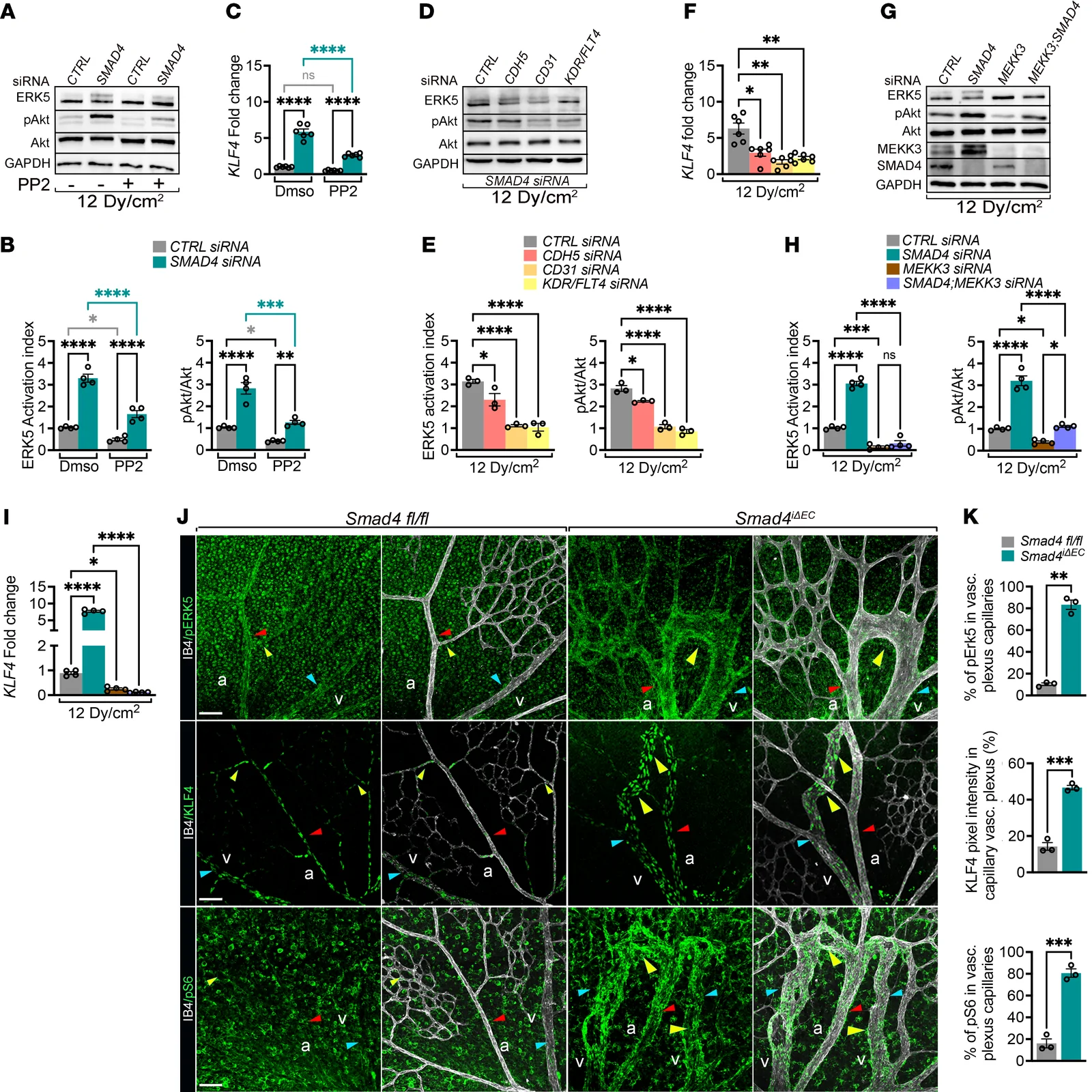
SMAD4 restricts junctional signals upstream of MEKK3/ERK5/KLF4/Akt activation. (**A**, **D**, and **G**) WB for the indicated proteins in HUVECs transfected with *CTRL* or *SMAD4* siRNAs subjected to 12 dynes/cm^2^ for 2 h and treated with DMSO or PP2 inhibitor (**A**) or transfected additionally with *CD31*, *CDH5*, and *KDR/FLT4* siRNAs (**D**) or with *MEKK3* siRNA (**G**). (**B**, **E**, and **H**) Quantification of ERK5 gel shift (upper band, quantified by densitometry) and of p-Akt normalized to total Akt, respectively (*n* = 4/group in **B** and **H**; *n* = 3/group in **E**). (**C**, **F**, and **I**) *KLF4* fold-change in the indicated genotypes and treatment conditions; *n* = 6/group in **C** and **F**; *n* = 4/group in **I**. (**J**) Colabeling of *Smad4^fl/fl^* and *Smad4*^iΔEC^ P6 retinas with p-ERK5 (green, upper panel), KLF4 (green, middle panel), pS6 (green, lower panel), and Isolectin B4 (IB4, white). Red, blue, and yellow arrowheads indicate arteries (a), veins (v), and capillaries, respectively. Scale bar: 50 μm. (**K**) Quantification of pERK5, KLF4, and pS6 in retina vascular plexus was calculated by normalizing fluorescence intensity threshold value per IB4^+^ vascular plexus capillary area (%) in *Smad4^fl/fl^* and *Smad4*^iΔEC^ neonates (*n* = 3 retinas per genotype). Data are represented as mean ± SEM. Two-way ANOVA (**B**, **C**, **E**, **F**, **H**, and **I**) and Student’s *t* test (**K**) were used to determine statistical significance. *P < 0.05, **P < 0.01, ***P < 0.005, ****P < 0.001.

**Figure 3:**
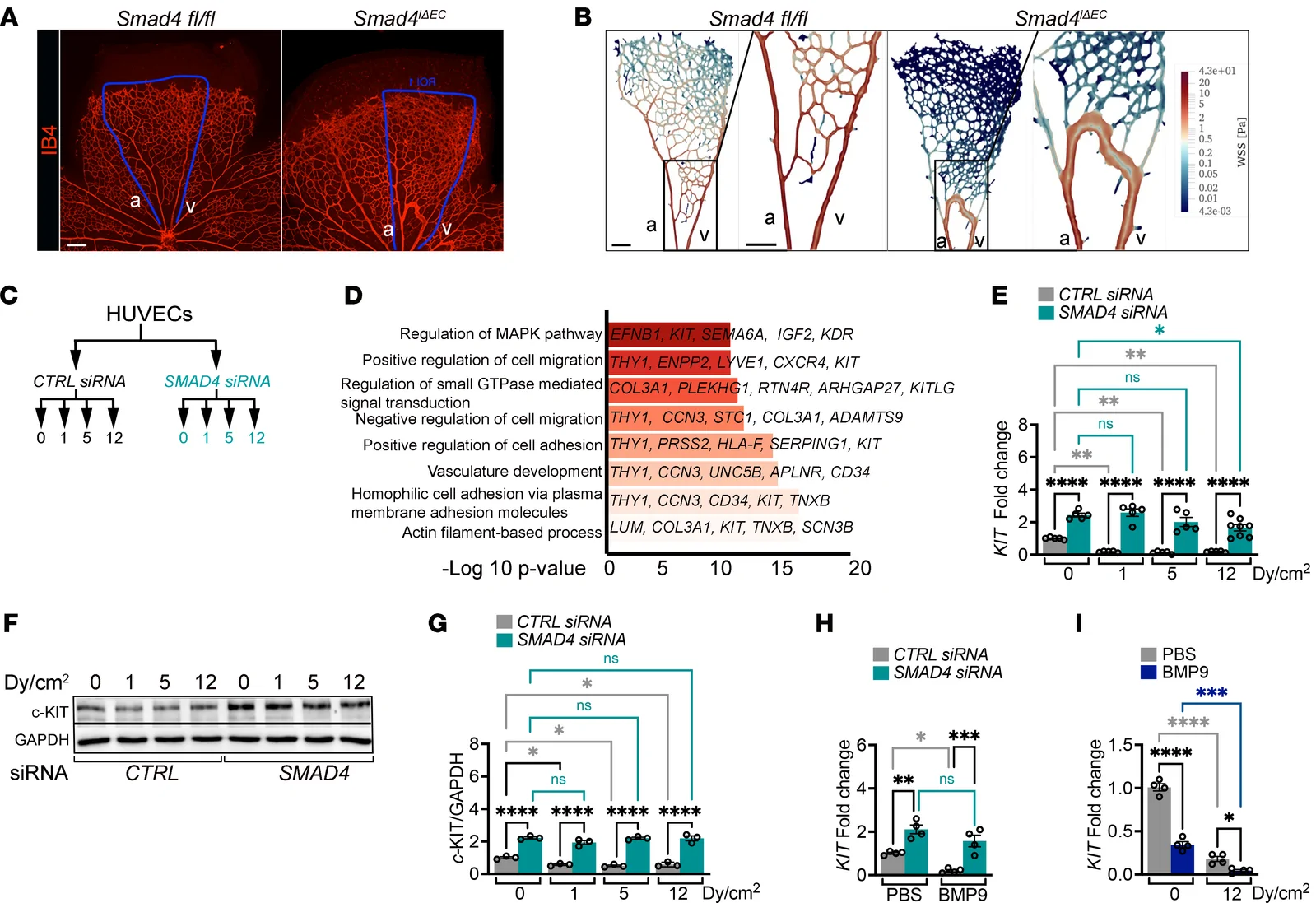
c-KIT upregulation characterizes the high-flow but low-FSS AVMs. (**A** and **B**) Whole-mount staining of IB4 (red) (**A**) and computer-simulated WSS modeling (**B**) of the *Smad4^fl/fl^* and *Smad4*^iΔEC^ P6 mouse retinas. Magnified vascular plexus (boxed) is shown in **B**. a, artery; v, vein. Scale bars: 100 μm (left) and 50 μm (right). (**C**) Experimental design of bulk RNA-seq in *CTRL* and *SMAD4* siRNA HUVECs grown statically or subjected to increasing magnitudes of 1-5-12 dynes/cm^2^ FSS for 2 h. (**D**) Significantly upregulated GO terms. (**E** and **F**) Expression of *KIT* by qPCR (**E**) and c-KIT protein by WB (**F**) in *CTRL* and *SMAD4* siRNA HUVECs grown statically or subjected to increasing magnitudes of 1-5-12 dynes/cm^2^ FSS for 2 h. (**G**) Quantification of c-KIT/GAPDH ratio (*n* = 3/group). (**H** and **I**) *KIT* fold-change in BMP9-stimulated (10 ng) *CTRL* and *SMAD4* siRNA HUVECs (*n* = 4/group) (**H**) and in HUVECs grown statically versus at 12 dynes/cm^2^ FSS for 2 h (*n* = 4/group) (**I**). Data are represented as mean ± SEM. Two-way ANOVA (**E** and **G**–**I**) was used to determine statistical significance. *P < 0.05, **P < 0.01, ***P < 0.005, ****P < 0.001.

**Figure 4:**
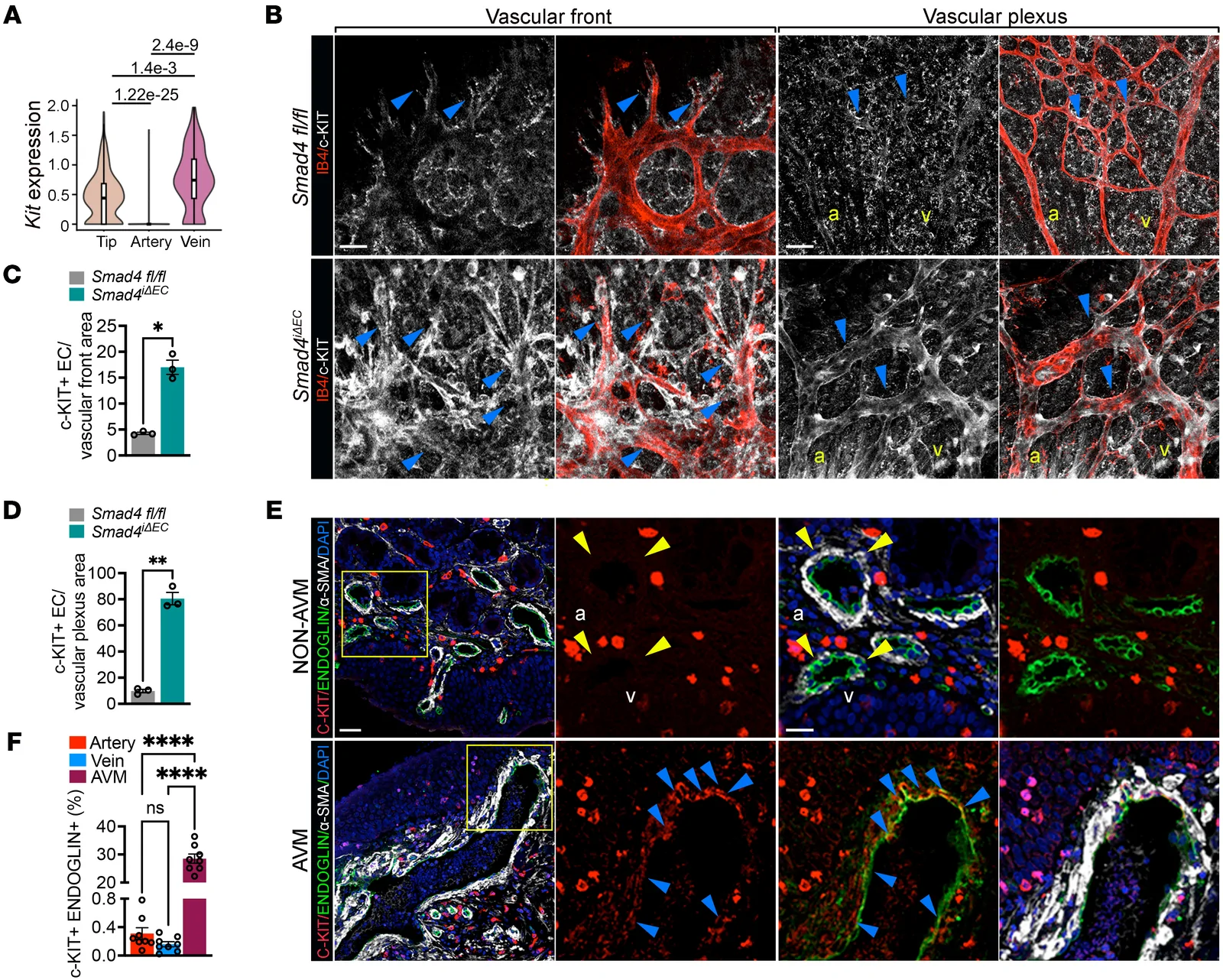
SMAD4 restricts c-KIT expression in vivo. (**A**) Reanalysis of *Kit* expression in scRNA-seq of P6 wild-type retinas (Tip, retina tip ECs). *P* values are above violin plots. (**B**) Colabeling for c-KIT (white) and IB4 (red) of retinal flatmounts from Tx-induced P6 *Smad4^fl/fl^* and *Smad4*^iΔEC^. Blue arrowheads point to c-KIT expression in tip cells (left panel) and vascular plexus (right panel). (**C** and **D**) Quantification of c-KIT fluorescence labeling intensity in ECs in the vascular front area (**C**) and in the vascular plexus (**D**) (*n* = 3 retinas/group). (**E**) Immunolabeling of human HHT2 patient nasal mucosa biopsy sections for c-KIT (red), ENDOGLIN (green), α-SMA (white), and DAPI (blue). Yellow arrowheads in non-AVM regions indicate ECs negative for c-KIT. Blue arrowheads in the insets indicate expression of c-KIT in the ECs in human AVM. (**F**) Quantification of c-KIT fluorescence labeling intensity in ENDOGLIN^+^ arterial versus venous endothelium (%), in non-AVM and AVM regions (*n* = 8, 4 pictures/patient biopsy). Scale bars: 50 μm in **B** and **E** left panels, 20 μm in insets in **E**. a, artery; v, vein. Data are represented as mean ± SEM. Bimod test was used in **A**; Student’s *t* test (**C** and **D**) and 1-way ANOVA (**F**) were used to determine statistical significance. *P < 0.05, **P < 0.01, ****P < 0.001.

**Figure 5:**
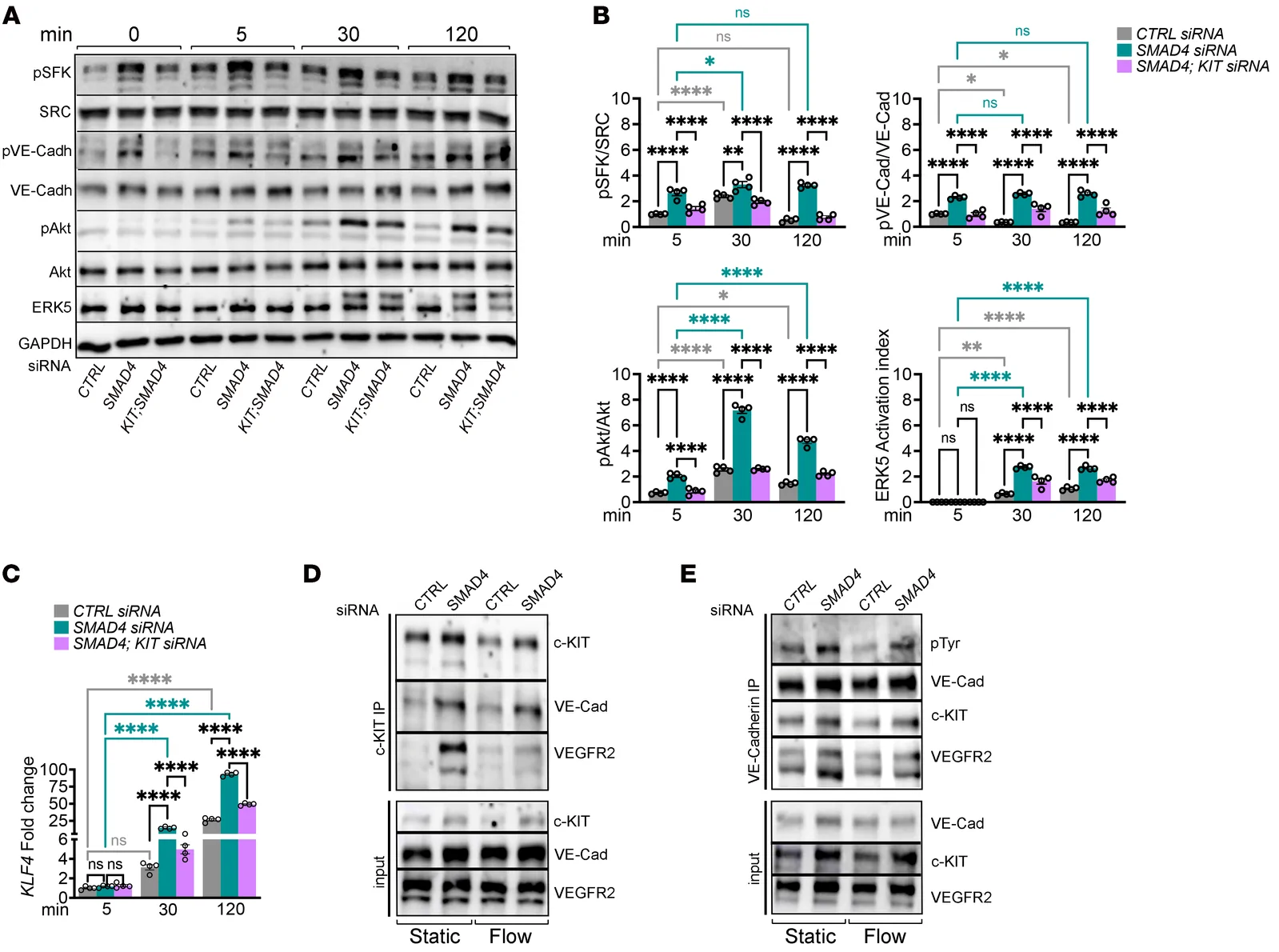
SMAD4 loss promotes c-KIT–dependent amplification of junctional flow signaling. (**A**) WB for the indicated proteins in HUVECs transfected with *CTRL*, *SMAD4*, and *SMAD4 KIT* siRNAs grown statically (0 minute) and 5-30-120 minutes 12 dynes/cm^2^ FSS. (**B**) Quantifications of activated SFK (Y418), VE-Cadherin (Y658), and Akt (S473) levels normalized to total SRC, VE-Cadherin, and Akt, respectively, and of ERK5 gel shift (upper band, quantified by densitometry) (*n* = 4/group). (**C**) *KLF4* fold-change in the indicated time points and conditions (*n* = 4/group). (**D** and **E**) *CTRL* versus *SMAD4* siRNA HUVECs grown statically and at 12 dynes/cm^2^ for 2 h were subjected to anti–c-KIT (**D**) and anti–VE-Cadherin (**E**) IP, and WB was performed for the indicated proteins. Data are represented as mean ± SEM. Two-way ANOVA (**B** and **C**) was used to determine statistical significance. *P < 0.05, **P < 0.01, ****P < 0.001.

**Figure 6:**
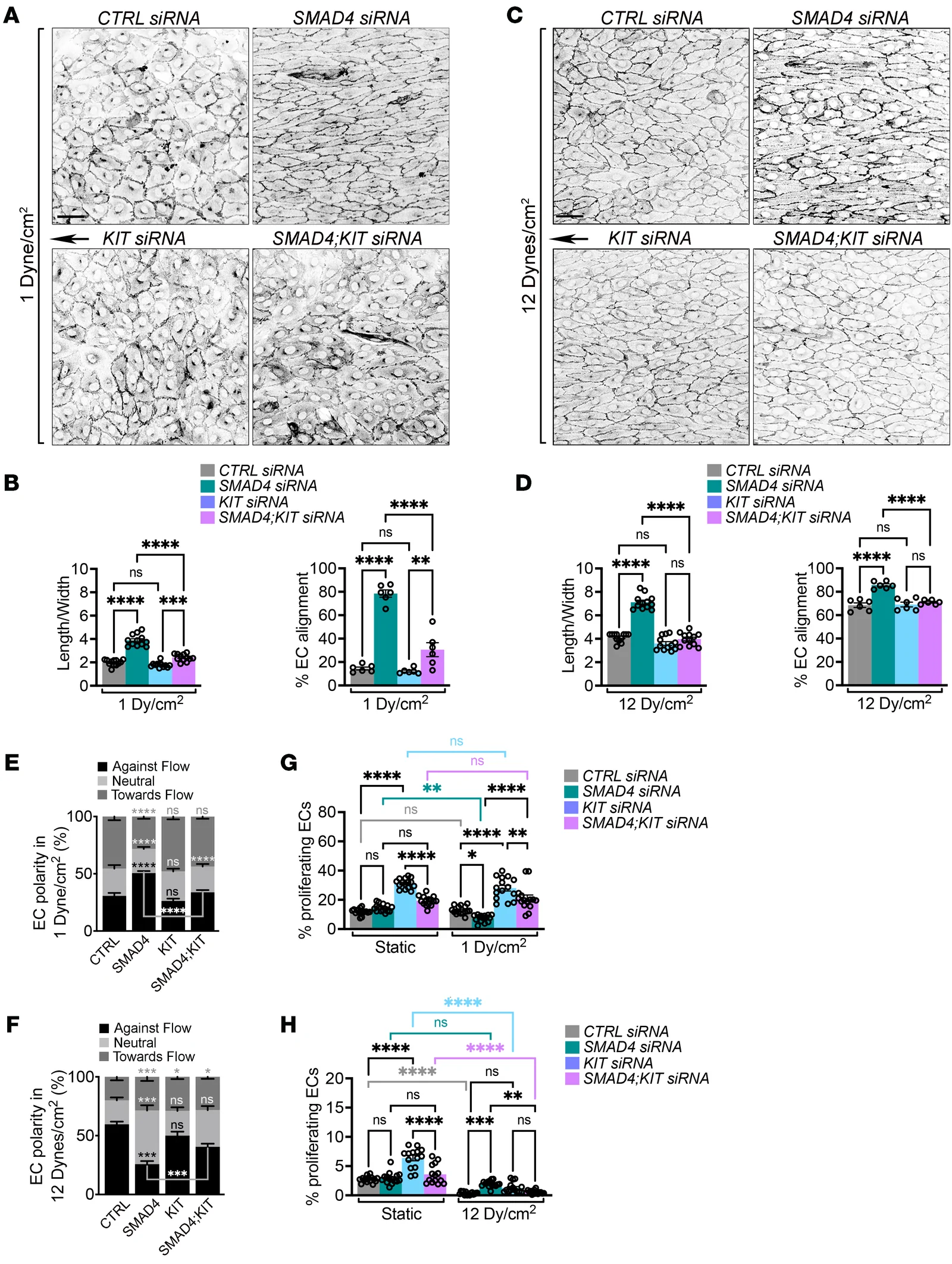
*KIT* inactivation in *SMAD4* KD cells restores the FSS set point for EC behaviors. (**A** and **C**) VE-Cadherin staining of *CTRL*, *KIT*, *SMAD4*, and *SMAD4 KIT* siRNA–treated HUVECs subjected to 1 dyne/cm^2^ (**A**) and 12 dynes/cm^2^ (**C**) for 48 h. Scale bars: 50 μm. Flow direction: right to left. (**B** and **D**) Quantification of length-to-width ratio (%) and of EC alignment parallel to flow direction (%) in the mentioned genotypes upon 1 dyne/cm^2^ (**B**) or 12 dynes/cm^2^ (**D**) (*n* = 12 average of 4 images [70–140 cells/image] per 3 independent experiments/group). (**E** and **F**) Quantification of EC polarization (%) against, toward the flow’s direction, or nonoriented (neutral) of HUVECs transfected with *CTRL*, *KIT*, *SMAD4*, and *SMAD4 KIT* siRNA subjected to 1 dyne/cm^2^ (**E**) and 12 dynes/cm^2^ (**F**) for 48 h (*n* = 15–18 images [50–100 cells/image] per 3 independent experiments/group). (**G** and **H**) S phase ratio (EdU^+^) per total number of DAPI^+^ nuclei (%) in *CTRL*, *KIT*, *SMAD4*, and *SMAD4 KIT* siRNA–treated HUVECs grown statically or subjected to 1 dyne/cm^2^ (**G**) and 12 dynes/cm^2^ (**H**) for 24 h (*n* = 15 images [200–250 cells/image] per 3 independent experiments/group). Data are represented as mean ± SEM. Two-way ANOVA (**B**–**H**) was used to determine statistical significance. *P < 0.05, **P < 0.01, ***P < 0.005, ****P < 0.001.

**Figure 7:**
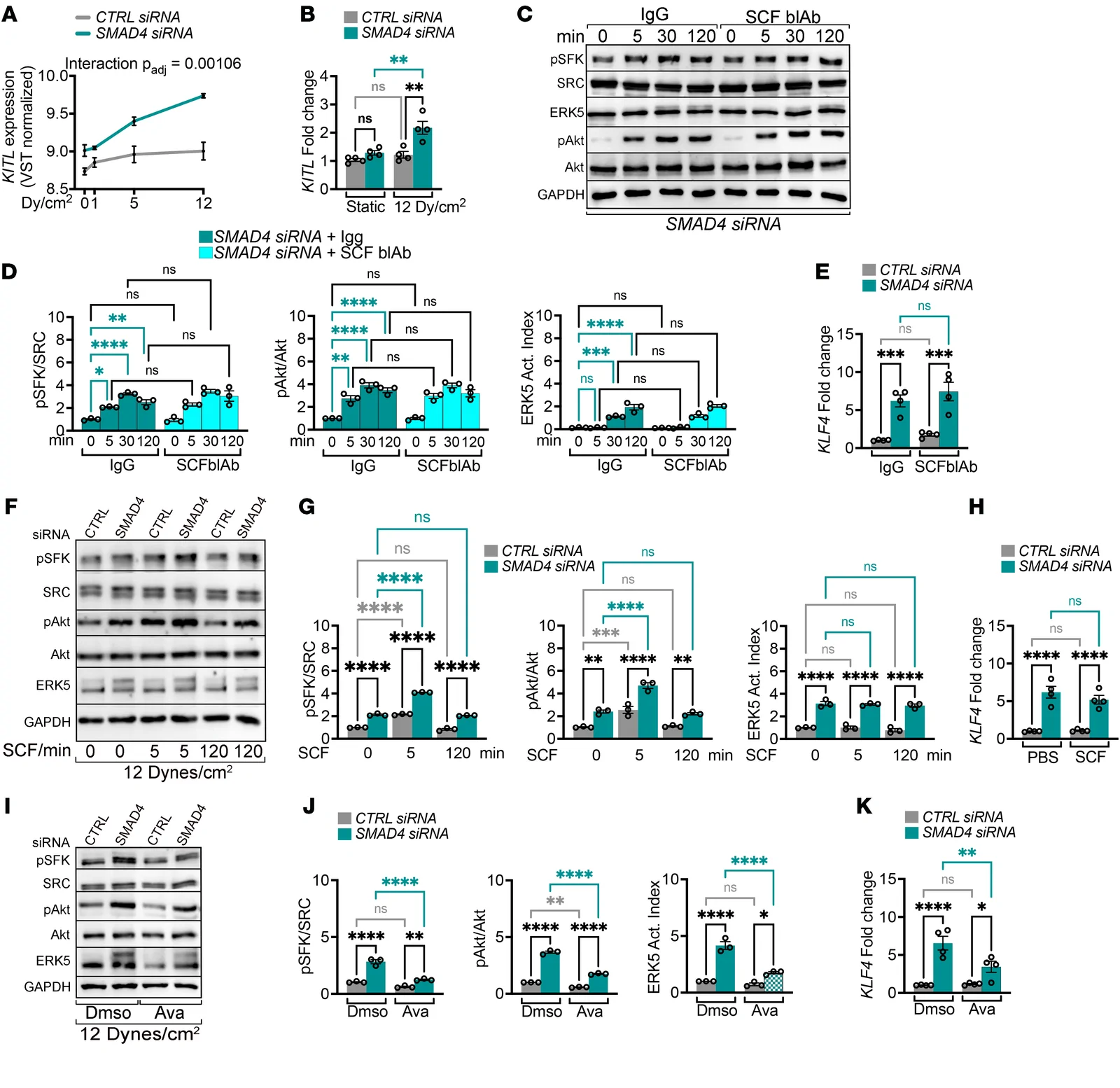
Ligand-independent c-KIT activation in *SMAD4* KD sustains flow signaling. (**A**) Relative *KITL* mRNA expression in RNA-seq groups (*n* = 3/group). (**B**) *KITL* qPCR in *CTRL* and *SMAD4* siRNA HUVECs grown statically versus 2 h of 12 dynes/cm^2^ (*n* = 4/group). (**C**) WB for the indicated proteins in *SMAD4* siRNA HUVECs grown statically or subjected to 12 dynes/cm^2^ for 5-30-120 minutes and treated with IgG or SCF blocking antibodies (SCF blAb). (**D**) Quantifications of SFK (Y418) and Akt (S473) levels normalized to total SRC and Akt, respectively, and of ERK5 gel shift (upper band, quantified by densitometry) (*n* = 3/group). (**E**, **H**, and **K**) *KLF4* qPCR in *CTRL* and *SMAD4* siRNA HUVECs treated with SCF blAb subjected to 12 dynes/cm^2^ FSS for 2 h (*n* = 4/group) (**E**) or treated with SCF for 2 h and subjected to 12 dynes/cm^2^ FSS (*n* = 4/group) (**H**) or subjected to 2 h of 12 dynes/cm^2^ treated with DMSO versus avapritinib (Ava) (*n* = 4/group) (**K**). (**F**) WB for the indicated proteins in *CTRL* and *SMAD4* siRNA HUVECs subjected to 12 dynes/cm^2^ for 2 h in the presence of SCF (50 ng/mL) for the indicated time (0 minute — no SCF; 5 and 120 minutes of SCF). (**G**) Quantifications of SFK (Y416) and Akt (S473) levels normalized to total SRC and Akt, respectively, and of ERK5 gel shift (*n* = 3/group). (**I**) WB for the indicated proteins in *SMAD4* siRNA HUVECs subjected to 12 dynes/cm^2^ for 2 h treated with DMSO or Ava. (**J**) Quantifications of SFK (Y416) and Akt (S473) levels normalized to total SRC and Akt, respectively, and of ERK5 gel shift (*n* = 3/group). Data are represented as mean ± SEM. Statistical significance for the interaction between siRNA groups and shear stress was determined by DESeq2 Likelihood Ratio Test (**A**). Two-way ANOVA (**B**, **D**, **E**, **G**, **H**, **J**, and **K**) was used to determine statistical significance. *P < 0.05, **P < 0.01, ***P < 0.005, ****P < 0.001.

**Figure 8:**
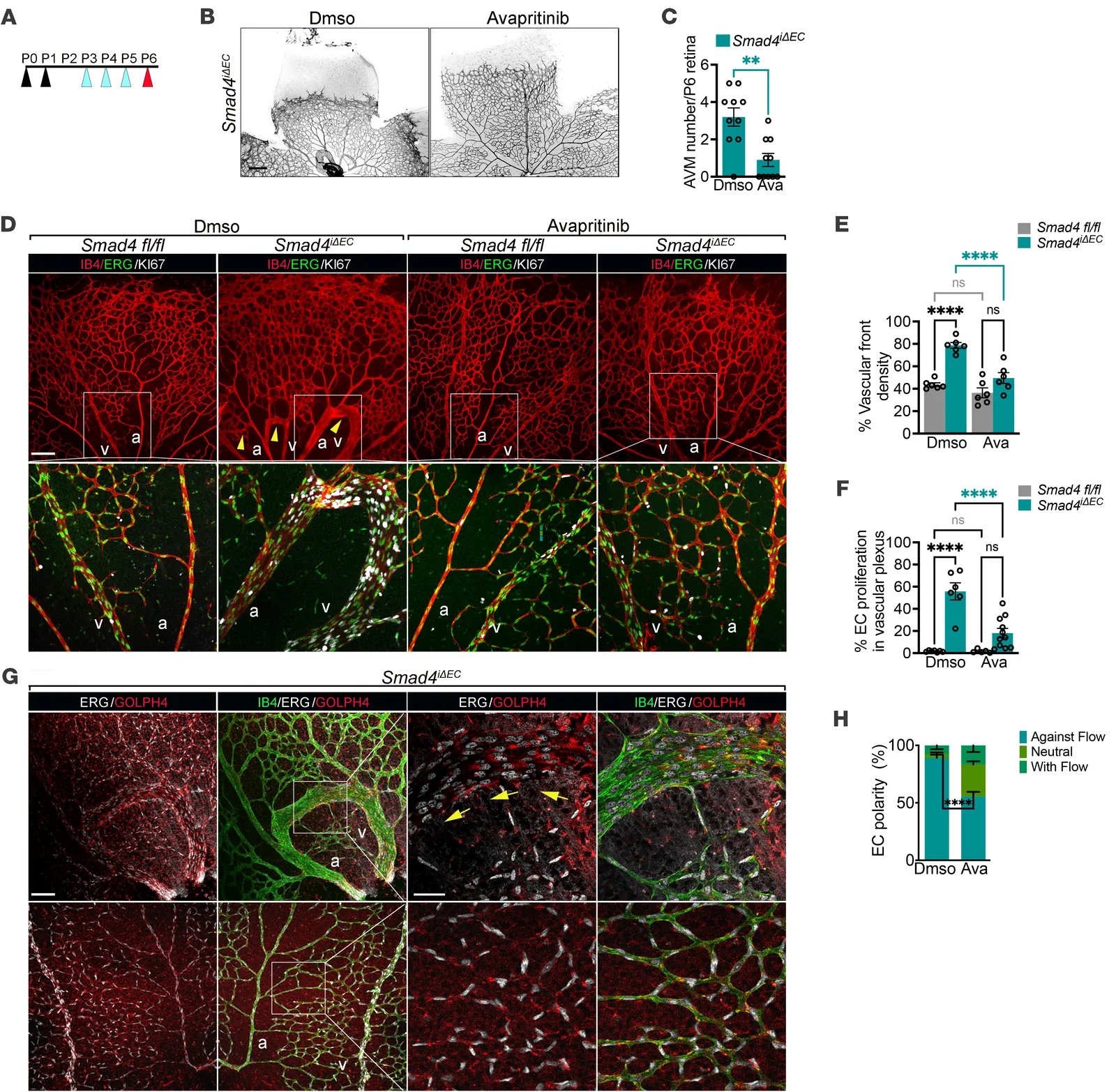
Inhibition of c-KIT kinase activity reverses AVM formation. (**A**) Schematic of Tx administration (black arrowheads), DMSO or Avapritinib (Ava) treatment (blue arrowheads), and harvesting (red arrowhead) in *Smad4^fl/fl^* and *Smad4*^iΔEC^ P6 neonates. (**B**) IB4 staining of *Smad4*^iΔEC^ P6 retinas, DMSO versus Ava treated. (**C**) Quantification of AVMs’ number per retina in *Smad4*^iΔEC^ DMSO- and Ava-treated neonates (*n* = 10 retinas/genotype). (**D**) Labeling for IB4 (red, upper panel) and IB4 (red), ERG (green), and KI67 (white) in lower panel of *Smad4^fl/fl^* and *Smad4*^iΔEC^ retinas from DMSO- and Ava-treated neonates. (**E**) Quantification of retinal front vascular density in DMSO- and Ava-treated neonates (*n* = 6 retinas/genotype). (**F**) Quantification of KI67^+^ERG^+^ per total ERG^+^ ECs in vascular plexus capillaries of *Smad4^fl/fl^* and *Smad4*^iΔEC^ retinas (*n* = 6–11 images from 3 retinas/group). (**G**) IB4 (green), ERG (white), and GOLPH4 (red) colabeling of P6 *Smad4*^iΔEC^ retina explants isolated from DMSO- and Ava-treated neonates. (**H**) Quantification of EC polarization: against, with flow, and neutral (nonpolarized) in capillaries from P6 *Smad4*^iΔEC^ retinas harvested from DMSO- and Ava-treated neonates (*n* = 2 images per retina from 3 retinas/group). Scale bars: 100 μm in **B**, 50 μm in **D** and **G**, and 20 μm in the insets in **D** and **G**. Yellow arrowheads in **D** indicate AVMs. Arrows in **G** indicate direction of EC migration. a, artery; v, vein. Data are represented as mean ± SEM. Student’s *t* test (**C** and **H**) and 2-way ANOVA (**E** and **F**) were used to determine statistical significance. **P < 0.01, ****P < 0.001.

## Version 1 10/01/2026

Electronic publication
