# Supplementary material for: Loss of Smad4 drives vascular malformations via c-KIT–dependent high–fluid shear stress mimicry
Source: J Clin Invest. 2026 Oct 1;136(19):e197609. doi: 10.1172/JCI197609 (PMC13626832; doi:10.1172/JCI197609)
Supplement: Supplementary file 1 [file jci-136-197609-s347.pdf]

SUPPLEMENTARY MATERIAL

Loss of *Smad4* drives vascular malformations via c-KIT-dependent high shear stress mimicry

Johannes Gahn[1*], Fan Wu[1*], Qing Zhang[1*], Yuxi Di[1], Tanmaya Behera[1], Yonggang Ren[2,3], Zohrah Hashemi[1], Kuheli Banerjee[1], Julio Cordero[2,3], Claudia Gherman[4], Kornelia Andorfer[5], Caroline Seebauer[6], Fatemeh Mirzapour-Shafiyi[7], Gergana Dobreva[2,3], Martin A. Schwartz[8], Roxana Ola[1#]

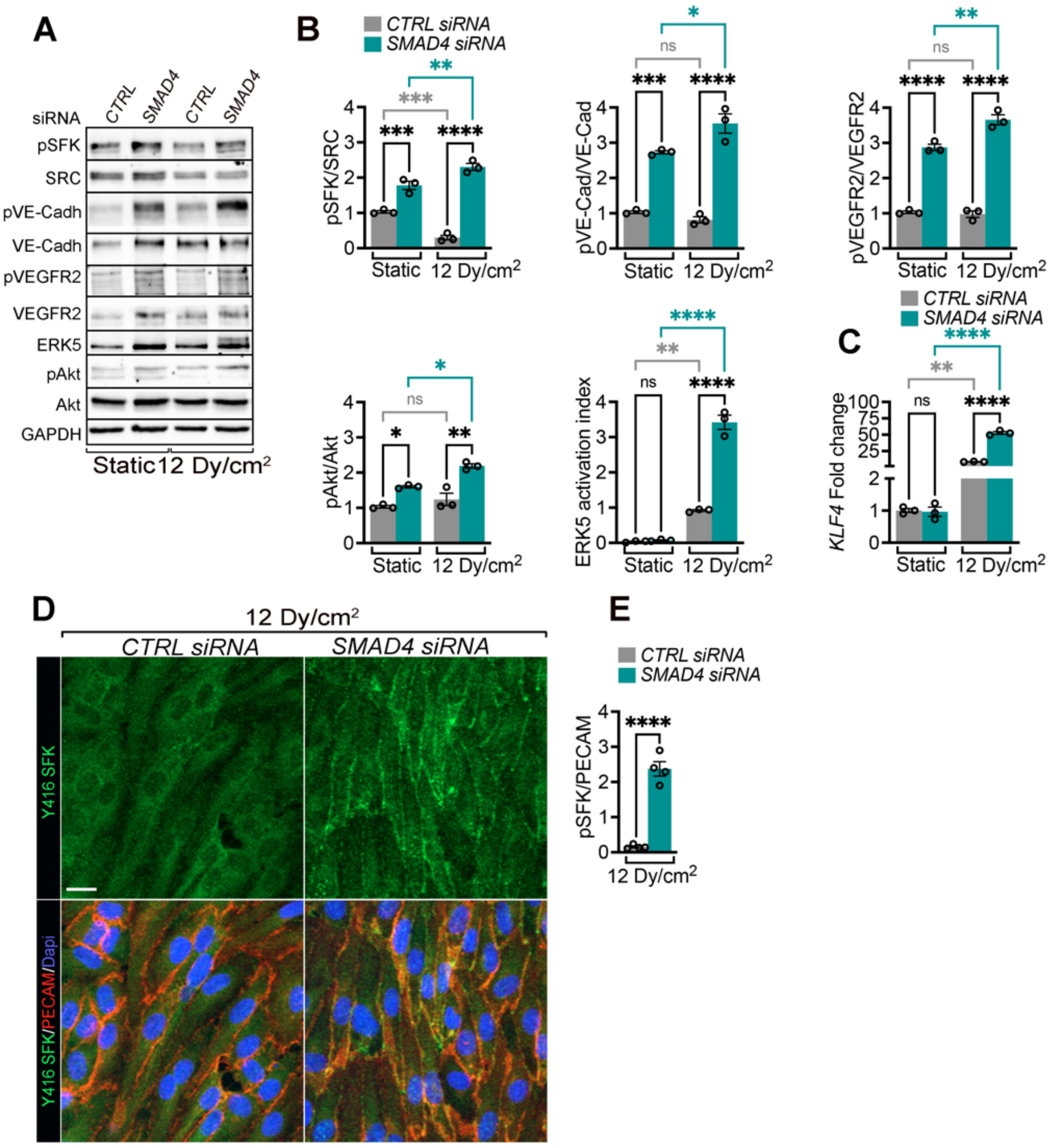

Supplementary Figure 1. ***SMAD4*****KD leads to sustained mechanotransduction in HUVECs**
(**A**) Representative immunoblots of the indicated proteins in *CTRL* and *SMAD4* siRNAs HUVECs grown in static versus 12 Dynes/cm^2 for 24 h (**B**) Quantifications of pSFK, pVE-Cadherin, pVEGFR2, and pAkt levels normalized to total SRC, VE-Cadherin, VEGFR2 and Akt, respectively and of ERK5 gel shift (n=3/group). (**C**) *KLF4* fold change in *CTRL* and *SMAD4* siRNAs HUVECs grown in static versus 12 Dynes/cm^2 FSS for 24 h (n=3/group). (**D**) Co-immunostaining for pSFK (green) and PECAM-1 (red) in *CTRL* and *SMAD4* siRNAs HUVECs subject to 12 Dynes/cm^2 conditions for 24 h. Scale bar: 50 µm. (**E**) Ratio of integrated intensity of junctional pSFK/PECAM (n=4 independent experiments). Data are represented as mean ± SEM. 2-way ANOVA in **B**,**C** and Student's t-test (**E**) were used to determine statistical significance. **P* <0.05, ***P* <0.01, ****P* <0.005, *****P* <0.001, ns=non-significant.

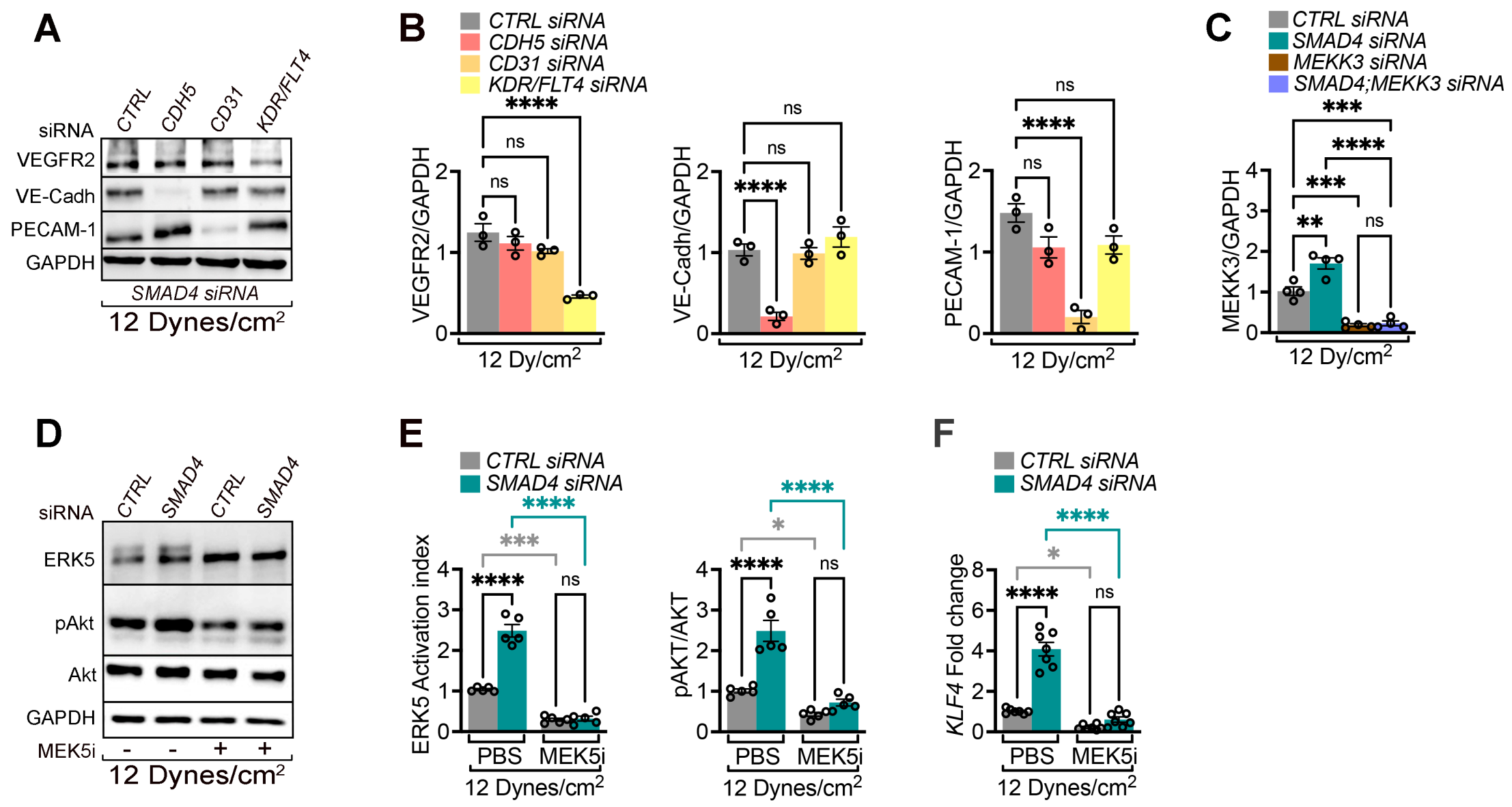

Supplementary Figure 2. Junctional mechanosensing apparatus acts upstream of MEKK3-MEK5-KLF4

(**A**) WB for the indicated proteins in *SMAD4* siRNA HUVECs subject to 12 Dynes/cm^2 for 2 h, transfected additionally with *CD31*, *CDH5* and *KDR/FLT4* siRNAs. (**B**) Quantifications of total levels of PECAM-1, VE-Cadherin and VEGFR2 normalized to GAPDH (n=3/group). (**C**) Quantifications of total levels of MEKK3 normalized to GAPDH (n=4/group). (**D**) WB for the indicated proteins in *CTRL* and *SMAD4* siRNAs HUVECs subjected to 12 Dynes/cm^2 for 2 h in the presence of PBS or MEK5 inhibitor (MEK5i). (**E**) Quantifications of ERK5 gel shift and Akt (S473) levels normalized to total Akt (n=5/group). (**F**) *KLF4* fold change in *CTRL* and *SMAD4* siRNAs HUVECs grown in static versus 12 Dynes/cm^2 FSS for 2 h treated with PBS versus MEK5i (n=7/group). Data are represented as mean ± SEM. 1-way ANOVA (**B**,**C**) and 2-way ANOVA (**E**,**F**) were used to determine statistical significance. **P* <0.05, ***P* <0.01, ****P* <0.005, *****P* <0.001, ns=non-significant.

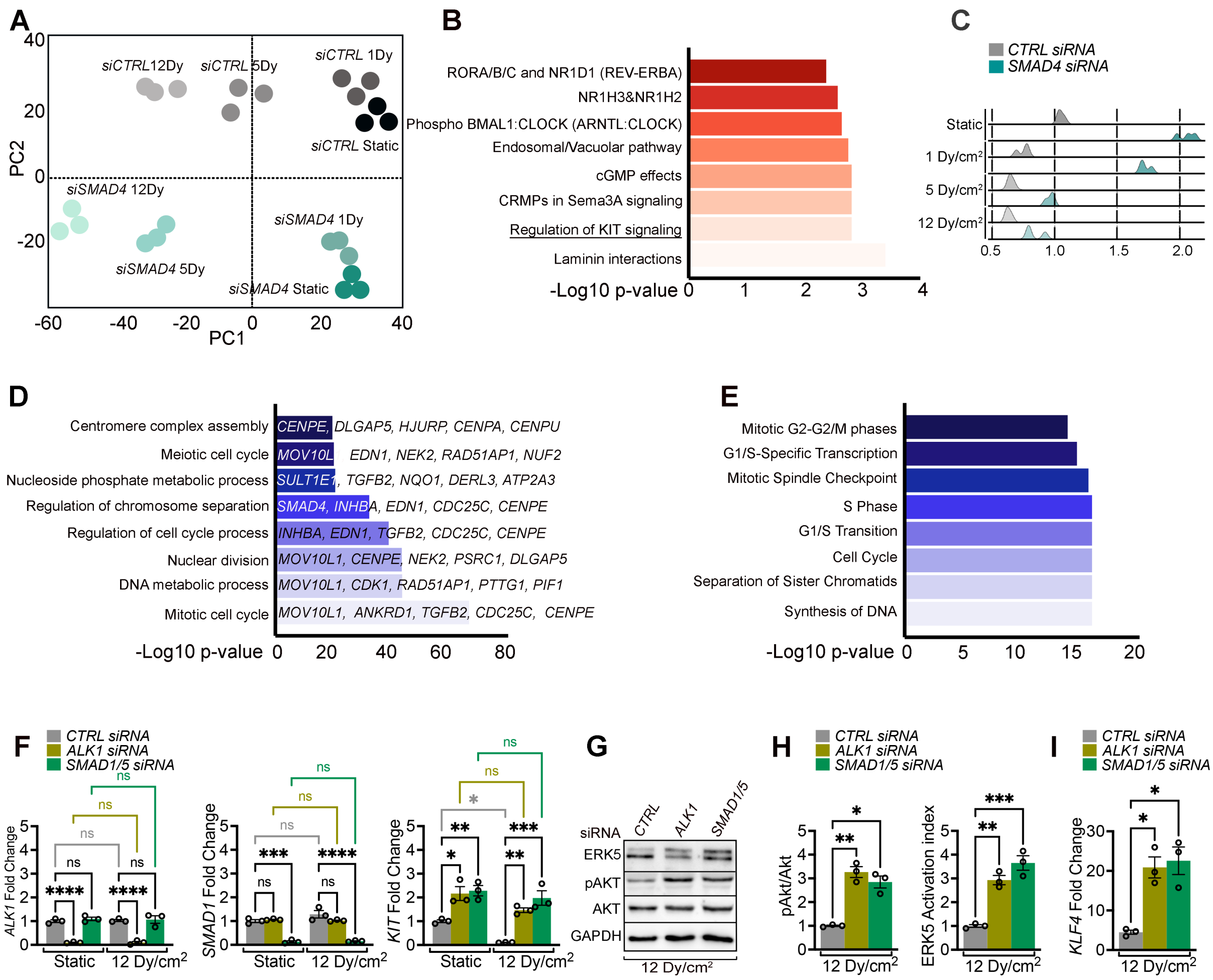

Supplementary Figure 3. ALK1-SMAD1/5 upstream of SMAD4 regulate c-KIT-mediated flow signaling

(**A**) Principal component analysis (PCA) of RNA seq. groups (n=3). (**B**) Reactome pathway analysis in *SMAD4* versus *CTRL* siRNA HUVECs in all conditions. (**C**) Expression of *KIT* in RNA seq. (Ridgeplot) (n = 3/group). (**D**,**E**) Significant downregulated GO terms (**D**) and reactome pathway (**E**) analysis in *CTRL* versus *SMAD4* siRNA conditions in RNA seq. (**F**) qPCR analysis for *ALK1*, *SMAD1* and *KIT* in HUVECs transfected with *CTRL, ALK1* and *SMAD1/5* siRNAs and grown in static versus 12 Dynes/cm^2 conditions for 2 h (n=3/group). (**G**) WB for the indicated proteins in *CTRL, ALK1* and *SMAD1/5* siRNAs HUVECs subject to 12 Dynes/cm^2 for 2 h. (**H**) Quantifications of ERK5 gel shift and activated Akt (S473) levels normalized to total Akt (n=3/group). (**I**) qPCR for *KLF4* in HUVECs transfected with *CTRL* versus *ALK1* and *SMAD1/5* siRNAs subject to 12 Dynes/cm^2 for 2 h. (n=3/group). Data are represented as mean ± SEM. 1-way ANOVA (**H**,**I**) and 2-way ANOVA (**F**) were used to determine statistical significance. *P <0.05, **P <0.01, ***P <0.005, ****P <0.001, ns=non-significant.

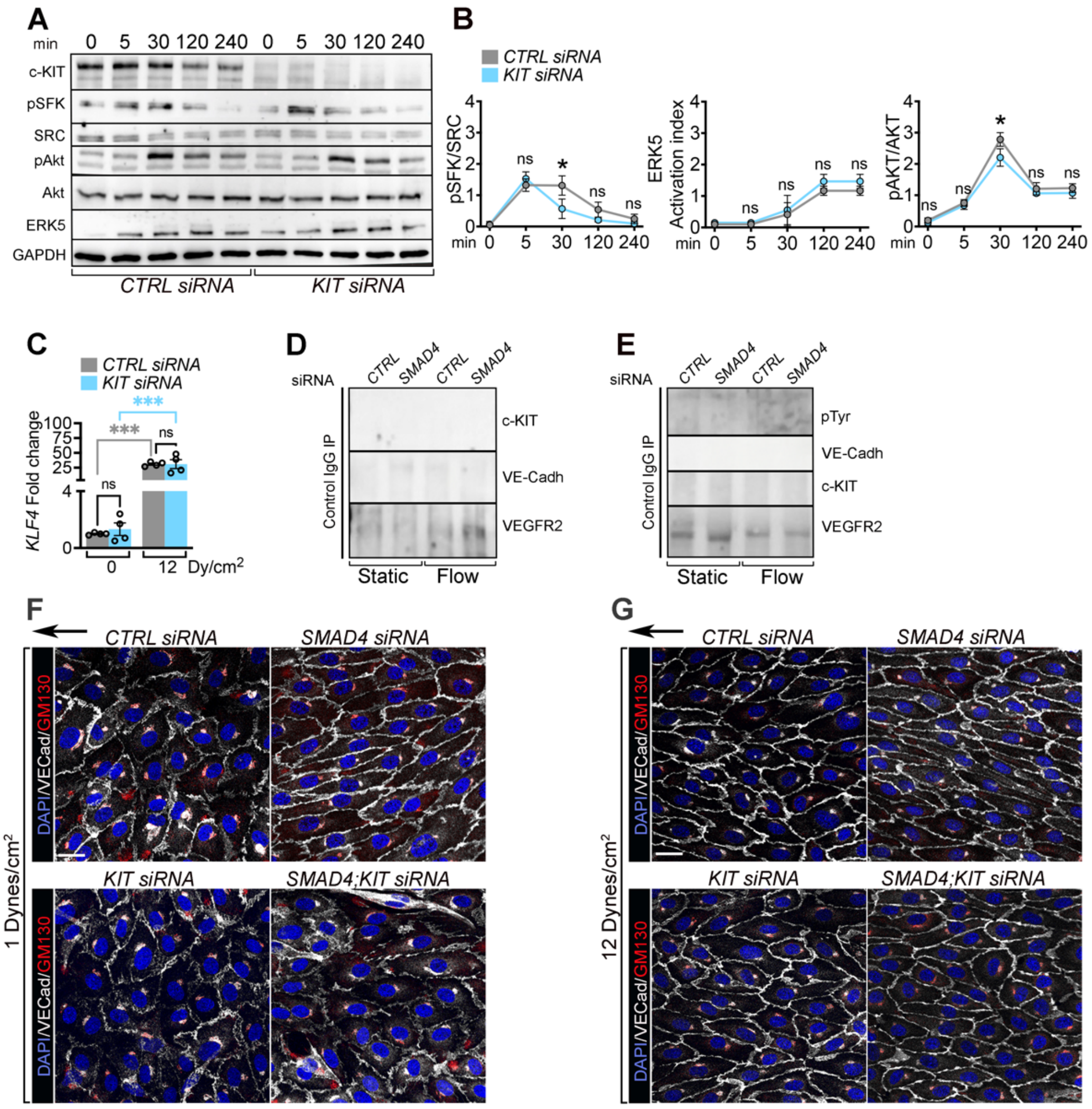

Supplementary Figure 4. c-KIT is dispensable for FSS mediated mechanotransduction
(**A**) WB for the indicated proteins in *CTRL* and *KIT* siRNAs HUVECs grown in static (0) or 12 Dynes/cm^2 for 5-30-120-240 min. (**B**) Quantifications of phosphorylated SFK (Y416), Akt (S473) levels normalized to total SRC and Akt, respectively and of ERK5 gel shift (n=3/group). (**C**) *KLF4* fold change in *CTRL* and *KIT* siRNAs HUVECs grown in static (0) versus 12 Dynes/cm^2 FSS for 2 h. (**D**,**E**) Rabbit immunoglobulin G (IgG) immunoprecipitation used as IP control for c-KIT (**D**) and VE-Cadherin (**E**). (**F**,**G**) Co-labeling for VE-Cadherin (white), GM130 (red), and DAPI (blue) of *CTRL*, *SMAD4, KIT* and *SMAD4;KIT* siRNAs HUVECs subject to 48 h 1 Dyne/cm^2 (**F**) or 12 Dynes/cm^2 (**G**). Black arrow indicates flow direction from right to left. Scale bars: 50 µm. Data are represented as mean ± SEM. Student's t-test (**B**) and 2-way ANOVA (**C**) were used to determine statistical significance. **P* <0.05, ****P* <0.005, *****P* <0.001, ns=non-significant.

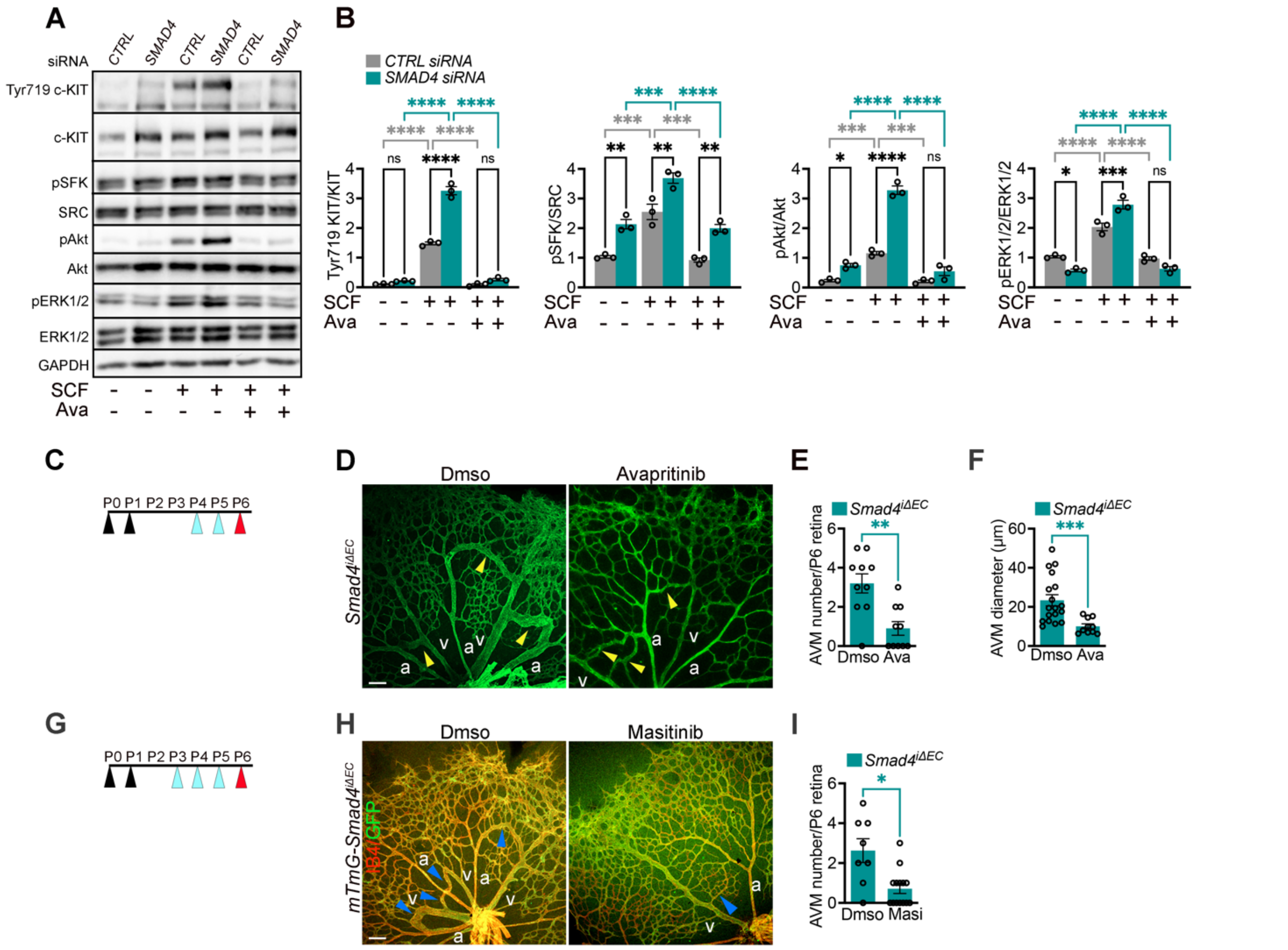

Supplementary Figure 5. Inhibition of c-KIT kinase activity improves AVM outcome

(**A**) WB for the indicated proteins in *CTRL* and *SMAD4* siRNAs HUVECs grown in static and stimulated with SCF (50ng/ml) for 5 min ± 1 μM Avapritinib (Ava). (**B**) Quantifications of phosphorylated c-KIT (Tyr719), SFK (Y416), Akt (S473) levels normalized to total c-KIT, SRC and Akt, respectively and of ERK5 gel shift (n=3/group). (**C**) Schematic of Tx administration (black arrowheads), Dmso or Ava treatment (blue arrowheads) and harvesting (red arrowheads) in *Smad4 fl/fl* and *Smad4*$^{\text{i}\Delta\text{EC}}$ P6 neonates. (**D**) IB4 labeling (green) of P6 retinas from *Smad4*$^{\text{i}\Delta\text{EC}}$ Dmso versus Avapritinib treated neonates at the indicated time points in **C**. (**E**,**F**) Quantification of AVMs' number per retina (n=6 retinas/genotype) and of capillary diameter in *Smad4*$^{\text{i}\Delta\text{EC}}$ P6 retinas (n=10-18 AVMs in 3 retinas/genotype) harvested from Dmso versus Ava treated neonates. (**G**) Schematic representation of Tx administration (black arrowheads), Dmso or Masitinib (Masi) treatment (blue arrowheads) and harvesting (red arrowheads) in *Smad4 fl/fl* and *Smad4*$^{\text{i}\Delta\text{EC}}$ P6 neonates. (**H**) Labeling for IB4 (red) and GFP (green) of P6 retinas *Smad4*$^{\text{i}\Delta\text{EC}}$ from Dmso versus Masitinib treated neonates. (**I**) Quantification of the number of AVMs per retina (n=8-14 retinas/genotype). Scale bar: 50 μm. Data are represented as mean ± SEM. 2-way ANOVA (**B**) and Student's t-test (**E**,**F**,**I**) were used to determine statistical significance. **P* <0.05, ****P* <0.005, *****P* <0.001, ns=non-significant

Supplemental Table 1. List of human primers

Primers	Forward Sequence	Reverse Sequence
KLF4	CCCACATGAAGCGACTTCCC	CAGGTCCAGGAGATCGTTGAA
KIT	CGTTCTGCTCCTACTGCTTCG	CCCACGCGGACTATTAAGTCT
KITL	AATCCTCTCGTCAAAACTGAAGG	CCATCTCGCTTATCCAACAATGA
GAPDH	CTGGGCTACACTGAGCACC	AAGTGGTCGTTGAGGGCAATG
SMAD4	QT00013174	QIAGEN
SMAD1	QT00103824	QIAGEN
ALK1	QT00050351	QIAGEN

Supplemental Table 2. List of primary antibodies used for immunoblotting

Primary Antibody	Dilution (IF)	Company	Cat. Number/Clone number
Isolectin GS-IB4 568	10 µg/ml	Thermo Fisher	I21412
Isolectin GS-IB4 488	10 µg/ml	Thermo Fisher	I21411
Isolectin GS-IB4 647	10 µg/ml	Thermo Fisher	I32450
c-Kit	1:100	Agilent	A450229-2 / Polyclonal
pErk5 (Thr218/Tyr220)	1:100	Cell Signaling	3371 / Polyclonal
PECAM-1	1:50	Santa Cruz	sc-376764/ H-3
Dapi	1 µg/mL	Sigma-Aldrich	5087410001
ENG	1:50	R&D Systems	AF1097 / Polyclonal
α-SMA	1:100	Agilent	M0851 / 1A4
KLF4	1:100	R&D Systems	AF3158 / Polyclonal
KI67 (eFluor™ 660)	1:100	Thermo Fisher	50-5698-82 / SolA15
ERG	1:100	Abcam	AB92513 / EPR3864
GOLPH4	1:200	Abcam	Ab28049 / Polyclonal
GM130	1:100	BD Pharmingen™	610823 / 35/GM130
VE-Cadherin	1:100	Cell Signaling	2500S / D87F2
Phospho-S6	1:100	Cell Signaling	5364S / D68F8

Supplemental Table 3. List of human siRNAs

siRNA	Company	Cat. Number
Control siRNA	Dharmacon/Horizon	D-001810-01
SMAD4 siRNA	Dharmacon/Horizon	L-003902-00
KIT siRNA	Dharmacon/Horizon	L-003150-00
MEKK3 siRNA	Dharmacon/Horizon	L-003301-00
CDH5 siRNA	Dharmacon/Horizon	L-003641-00
CD31 siRNA	Sigma	GGCCCCAAUACACUUCACA<br>AACCACUGCAGAGUACCAG
KDR siRNA	Dharmacon/Horizon	L-003148-00
FLT4 siRNA	Sigma	GAAGCCCAAUCAAUAACUGUU<br>UAAGCACUCUGGCAGAGAAUU
SMAD1 siRNA	Dharmacon/Horizon	L-012723-00
SMAD5 siRNA	Dharmacon/Horizon	L-015791-00
ACVRL1 siRNA	Dharmacon/Horizon	L-005302-02

Supplemental Table 4. List of chemicals

Inhibitor/recombinant Protein	Company	Cat. Number
PP2	Sigma-Aldrich	529576
BIX02189	Selleck Chemicals	S1531
Masitinib mesylate	MedChemExpress	HY-10209A
Avapritinib (BLU-285)	MedChemExpress	S8553
Human recombinant SCF	Thermo Fisher	AF-300-07
Human SCF blocking antibody	R&D Systems	AF-255-NA

Supplemental Table 5. List of primary antibodies used in WB

Primary Antibody	Dilution (WB)	Company	Cat. Number/Clone number
pSFK (Tyr416)	1:2000	Cell Signaling	6943 / D49G4
SRC	1:2000	Cell Signaling	2109 / 36D10
pVE-Cadherin (Tyr658)	1:1000	Abcam	ab119785 / Polyclonal
VE-Cadherin	1:1000	Cell Signaling	2500S / D87F2
pVEGFR2 (Tyr1054)	1:1000	Thermo Fisher	44-1047G / Polyclonal
VEGFR2	1:1000	Cell Signaling	2479 / 55B11
ERK5	1:1000	Cell Signaling	3372 / Polyclonal
pAKT (S473)	1:2000	Cell Signaling	4060 / D9E
AKT	1:2000	Cell Signaling	4685 / 11E7
GAPDH	1:5000	Cell Signaling	5174 / D16H11
MEKK3	1:1000	Cell Signaling	5727 / Polyclonal
c-KIT	1:1000	Agilent	A450229-2 / Polyclonal
c-KIT (Tyr719)	1:1000	Cell Signaling	3391 / Polyclonal
SMAD4	1:1000	Cell Signaling	38454 / D3M6U
p44/42 (ERK1/2)	1:1000	Cell Signaling	4695 / 137F5
Phospho-p44/42 (P-ERK1/2)	1:1000	Cell Signaling	4370S / D13.14.4E

Supplemental Table 6. List of secondary antibodies used in WB and immunostaining

Secondary Antibody	Dilution	Company	Cat. Number
anti-rabbit HRP	1:5000	Vector Laboratories	PI-1000-1
anti-mouse HRP	1:5000	Vector Laboratories	PI-2000
anti-Rabbit 488	1:250	Thermo Fisher	A-21206
anti-Goat 488	1:250	Thermo Fisher	A-11055
anti-Mouse 568	1:250	Thermo Fisher	A-10037
anti-Rabbit 568	1:250	Thermo Fisher	A-10042
anti-Mouse 647	1:250	Thermo Fisher	A-31571
anti-Rabbit 647	1:250	Thermo Fisher	A-31573
