# Supplementary material for: Loss of Smad4 drives vascular malformations via c-KIT–dependent high–fluid shear stress mimicry
Source: J Clin Invest. 2026 Oct 1;136(19):e197609. doi: 10.1172/JCI197609 (PMC13626832; doi:10.1172/JCI197609)

Full unedited blot/gel for Figure 1 A

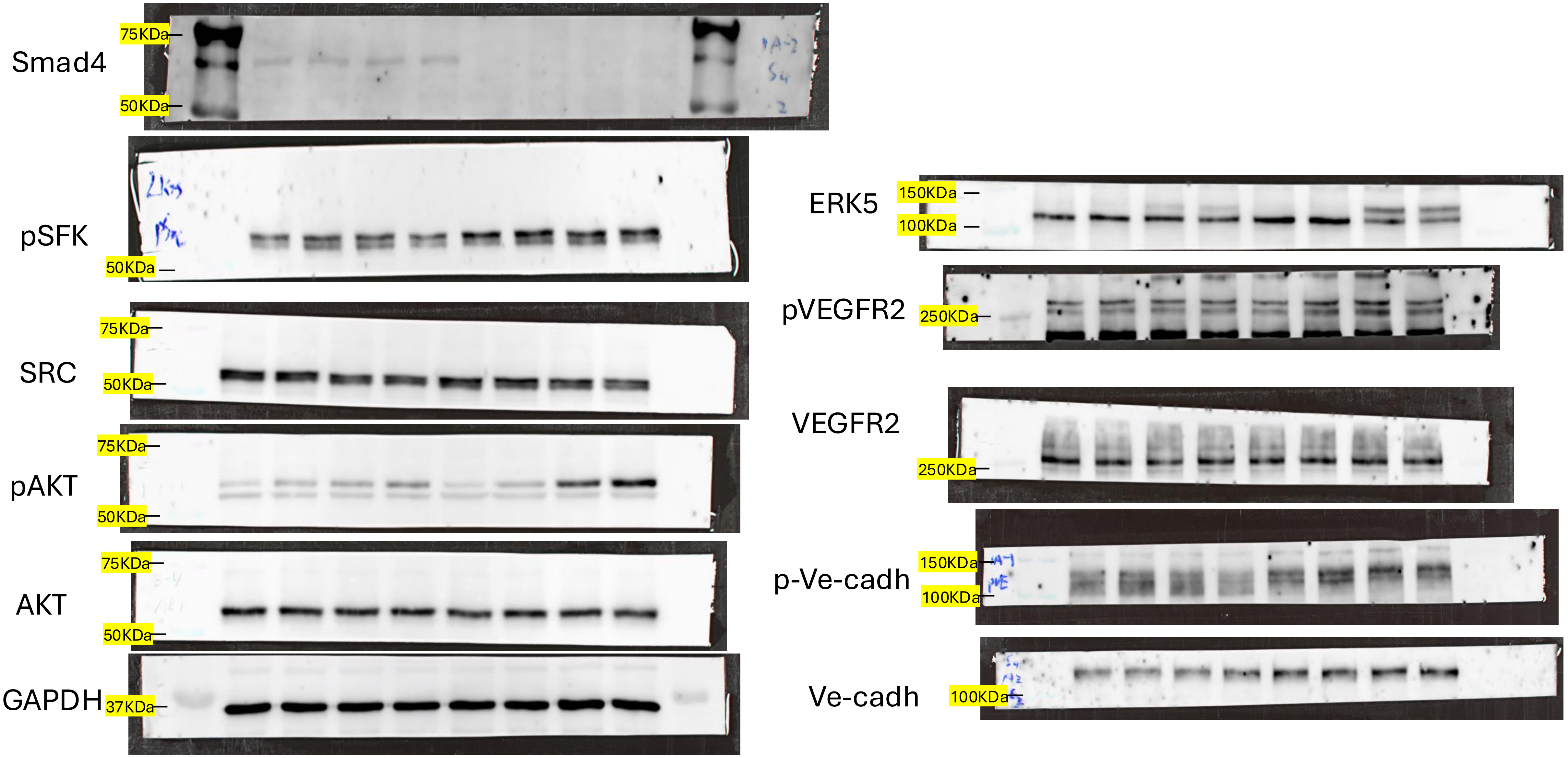

Full unedited blot/gel for Figure 1 C

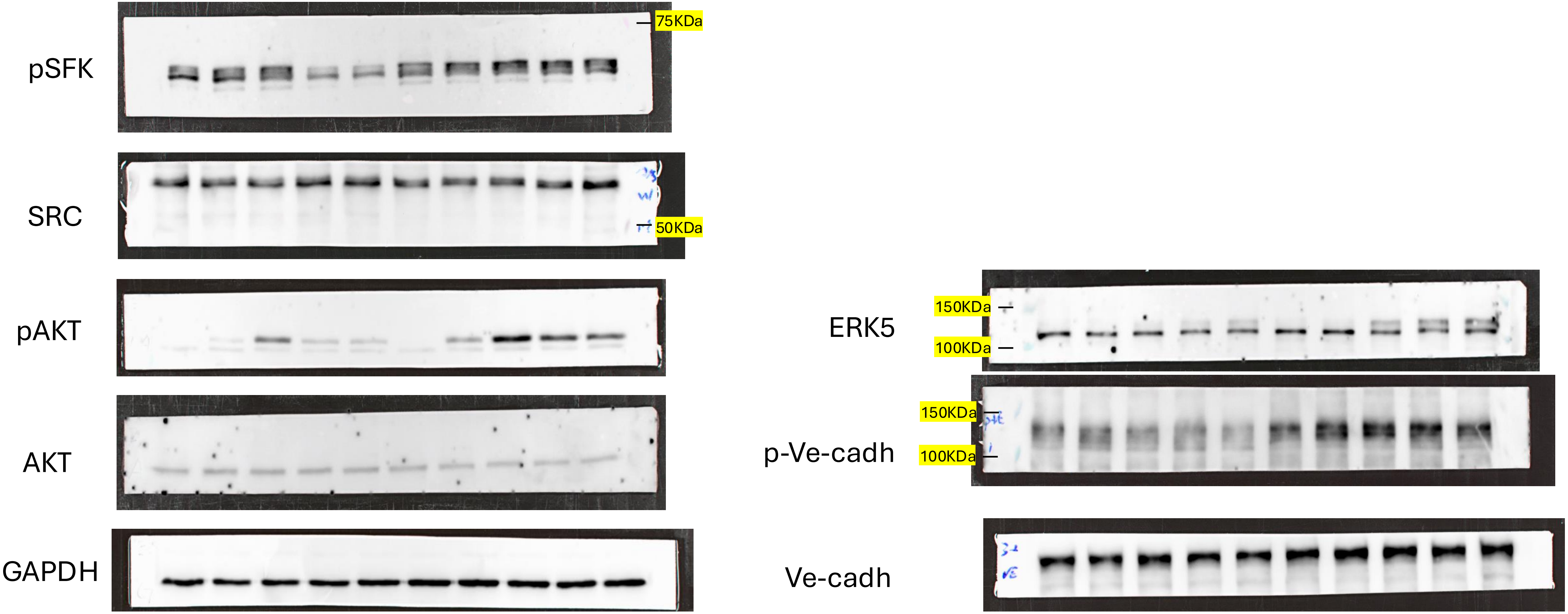

Full unedited blot/gel for Figure 2 A

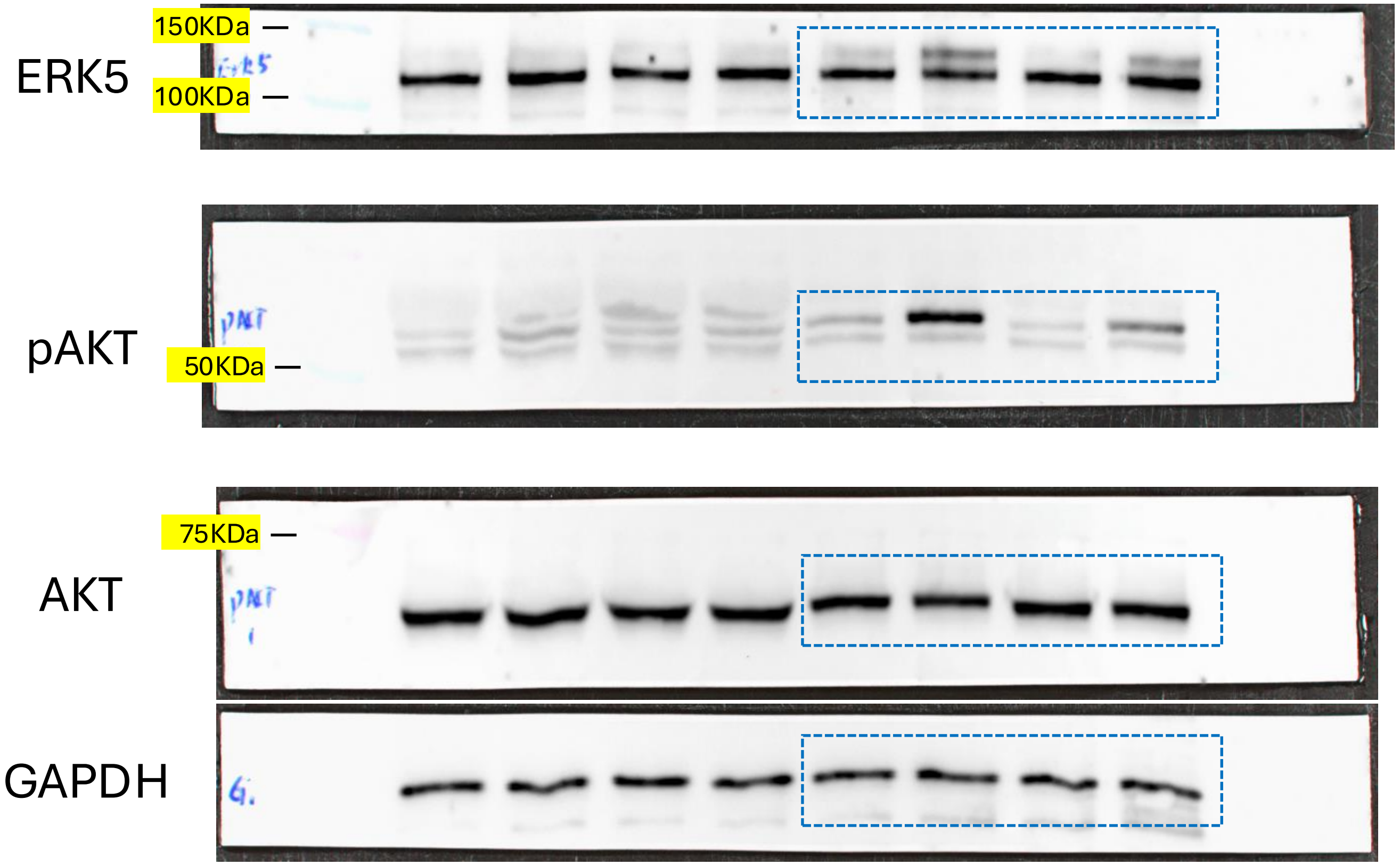

Full unedited blot/gel for Figure 2D

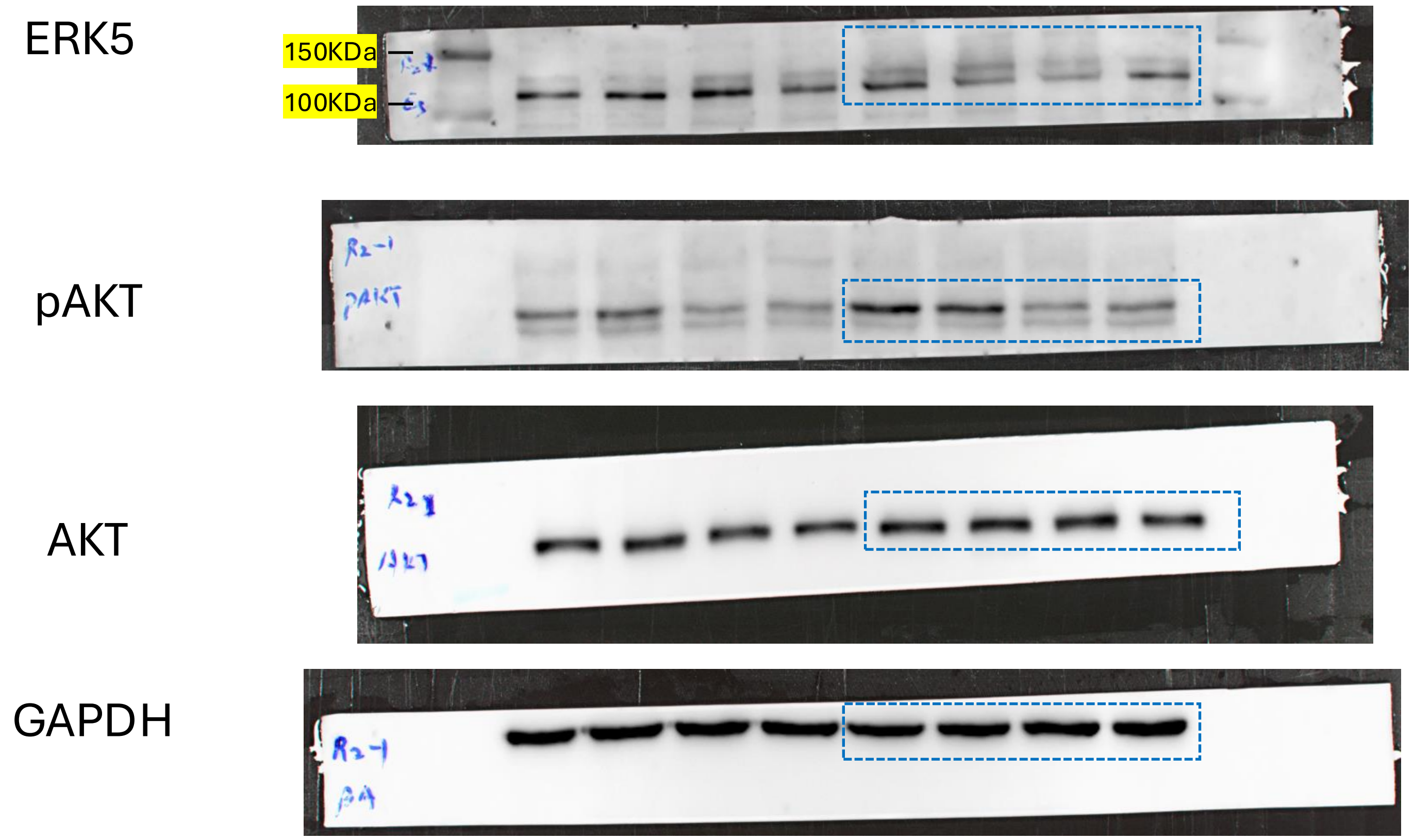

Full unedited blot/gel for Figure 2G

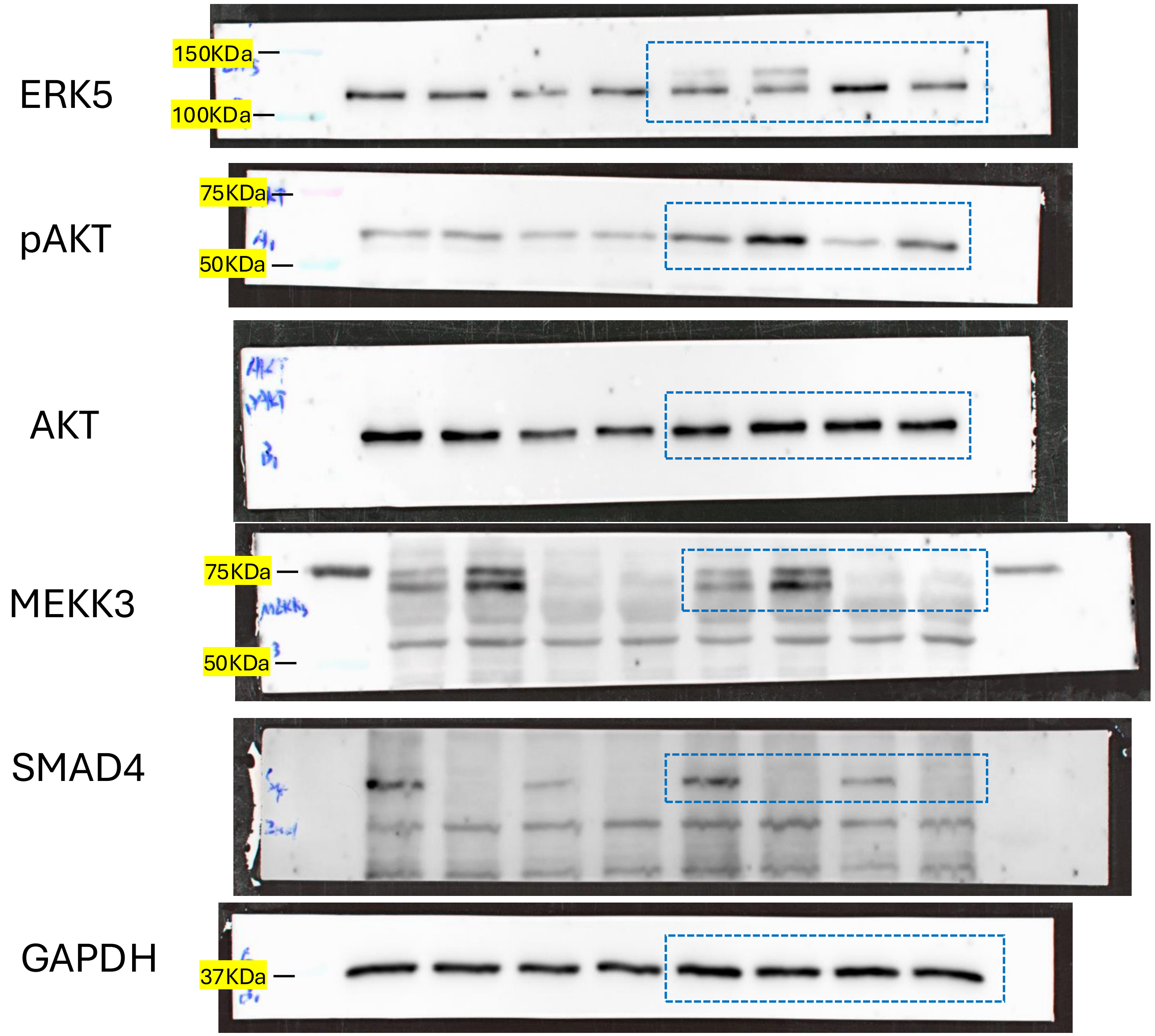

Full unedited blot/gel for Figure 3F

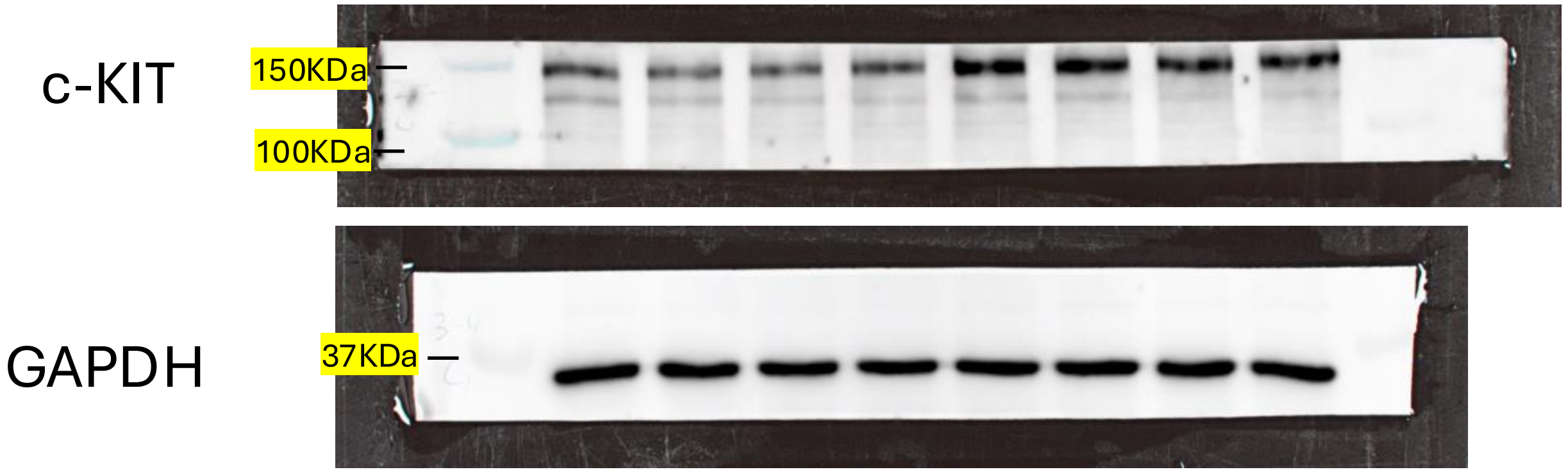

Full unedited blot/gel for Figure 5A

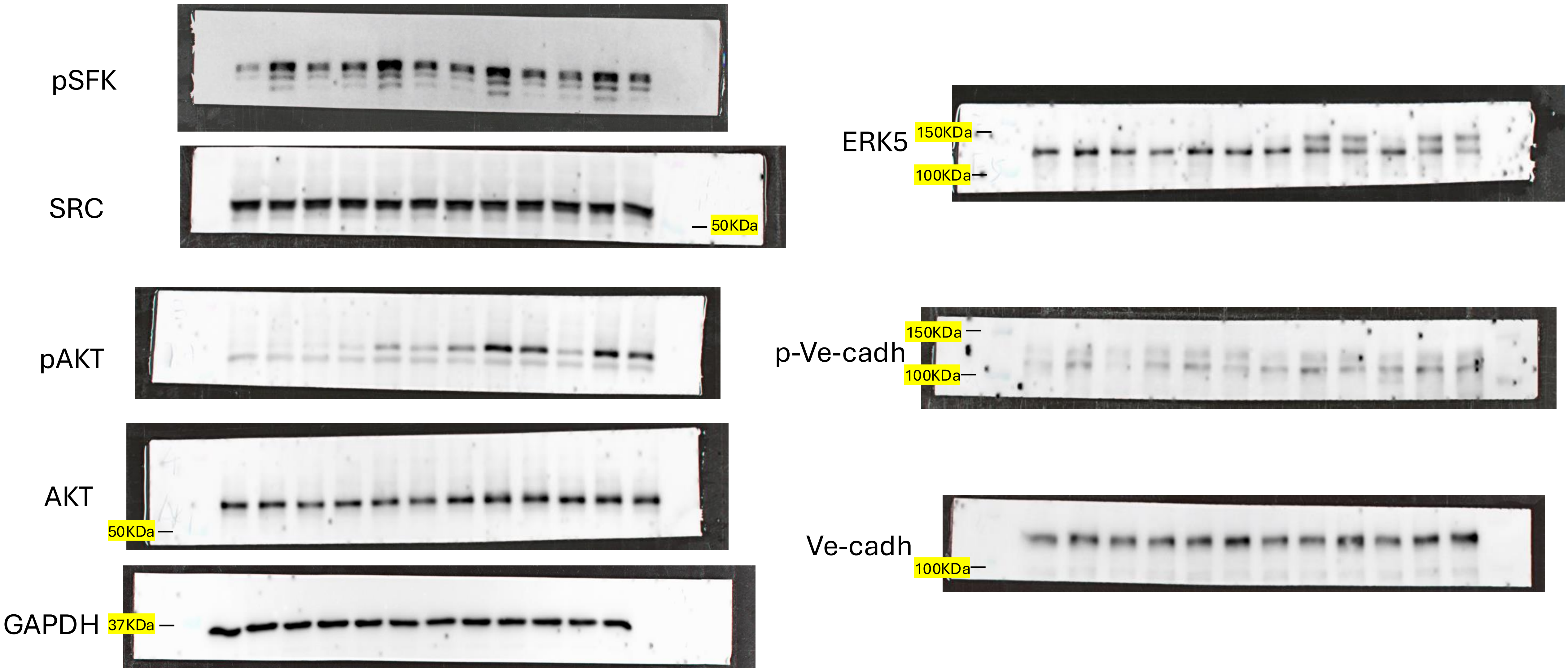

Full unedited blot/gel for Figure 5D

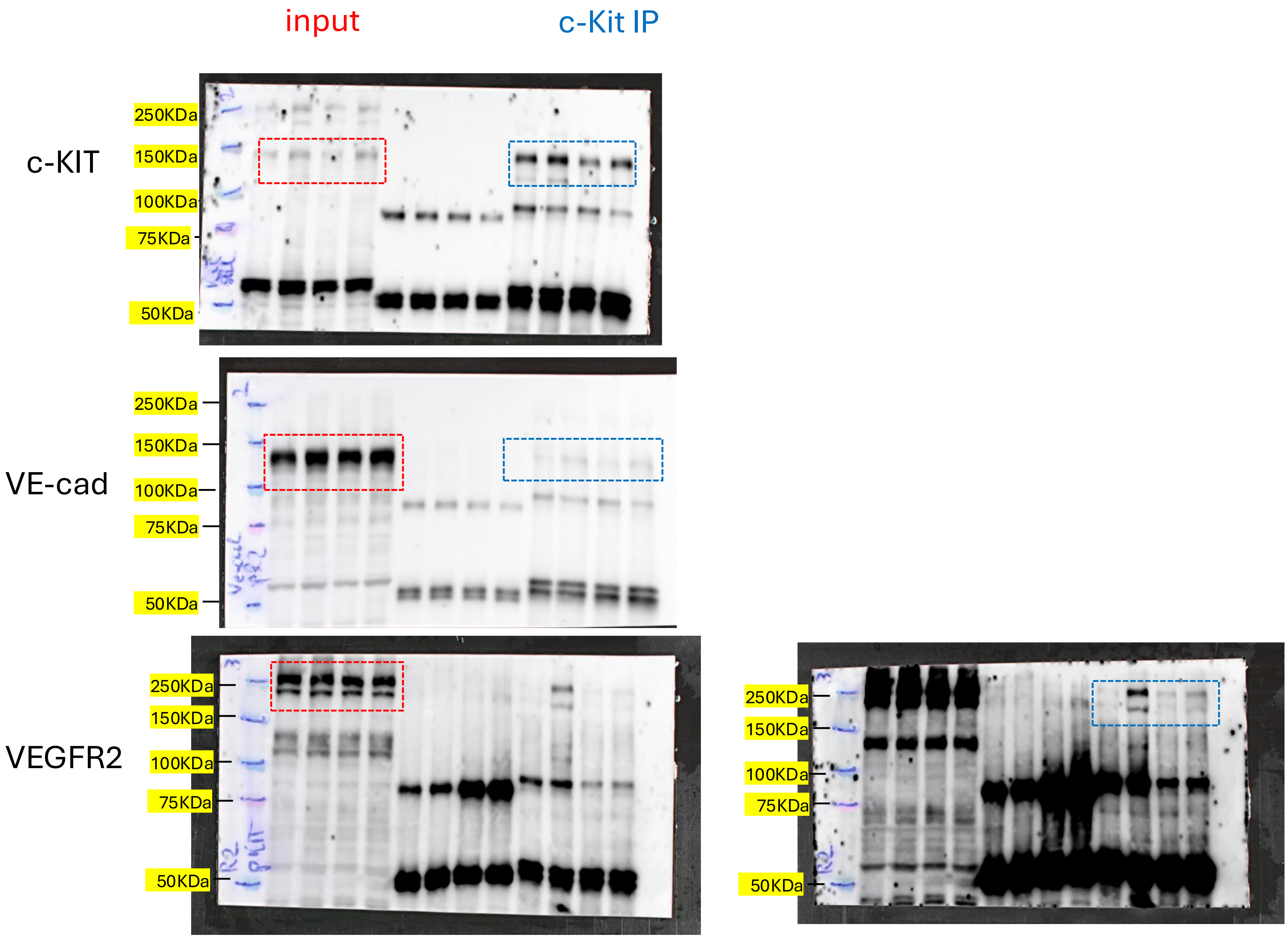

Full unedited blot/gel for Figure 5E

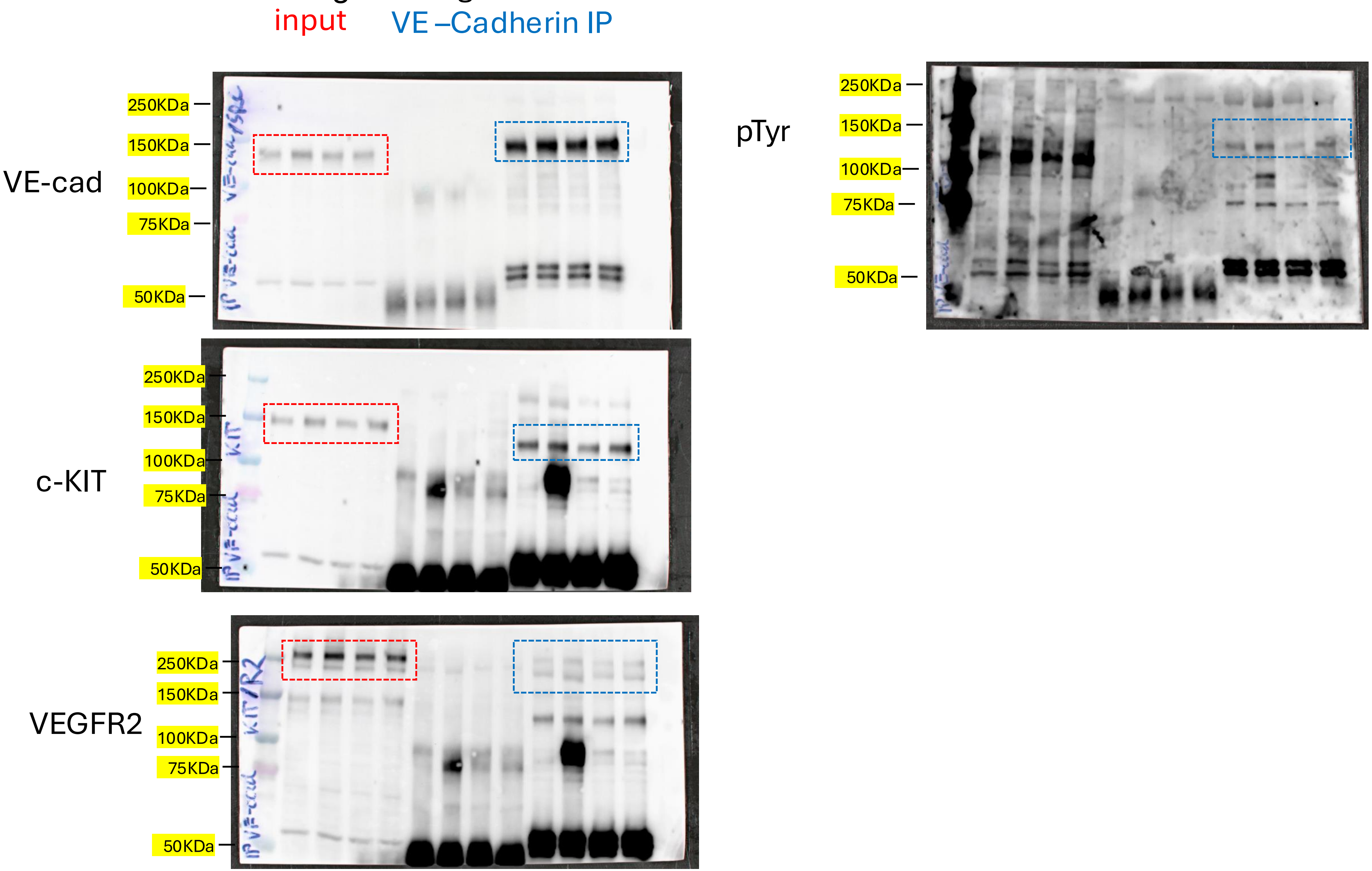

Full unedited blot/gel for Figure 7C

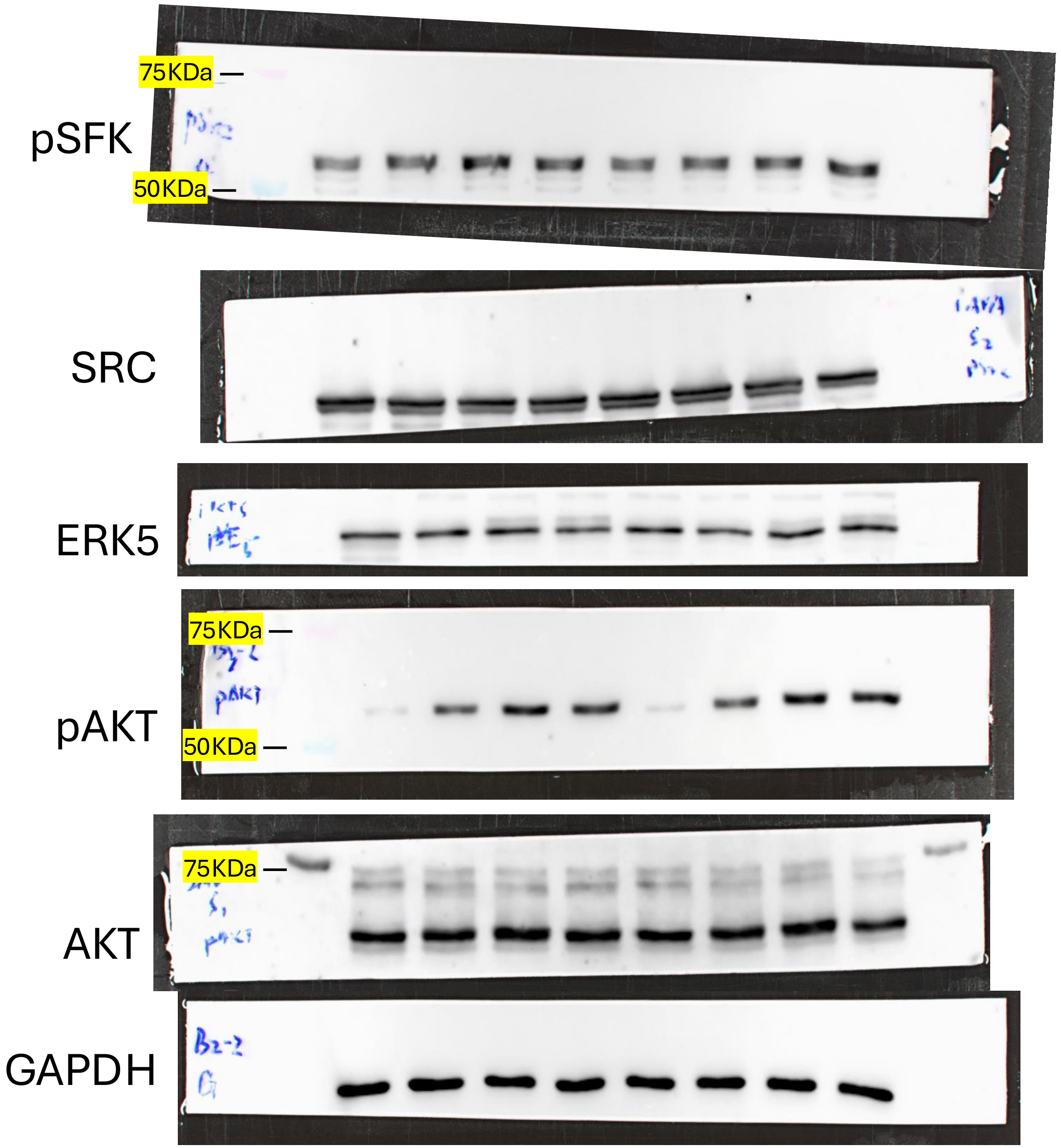

Full unedited blot/gel for Figure 7F

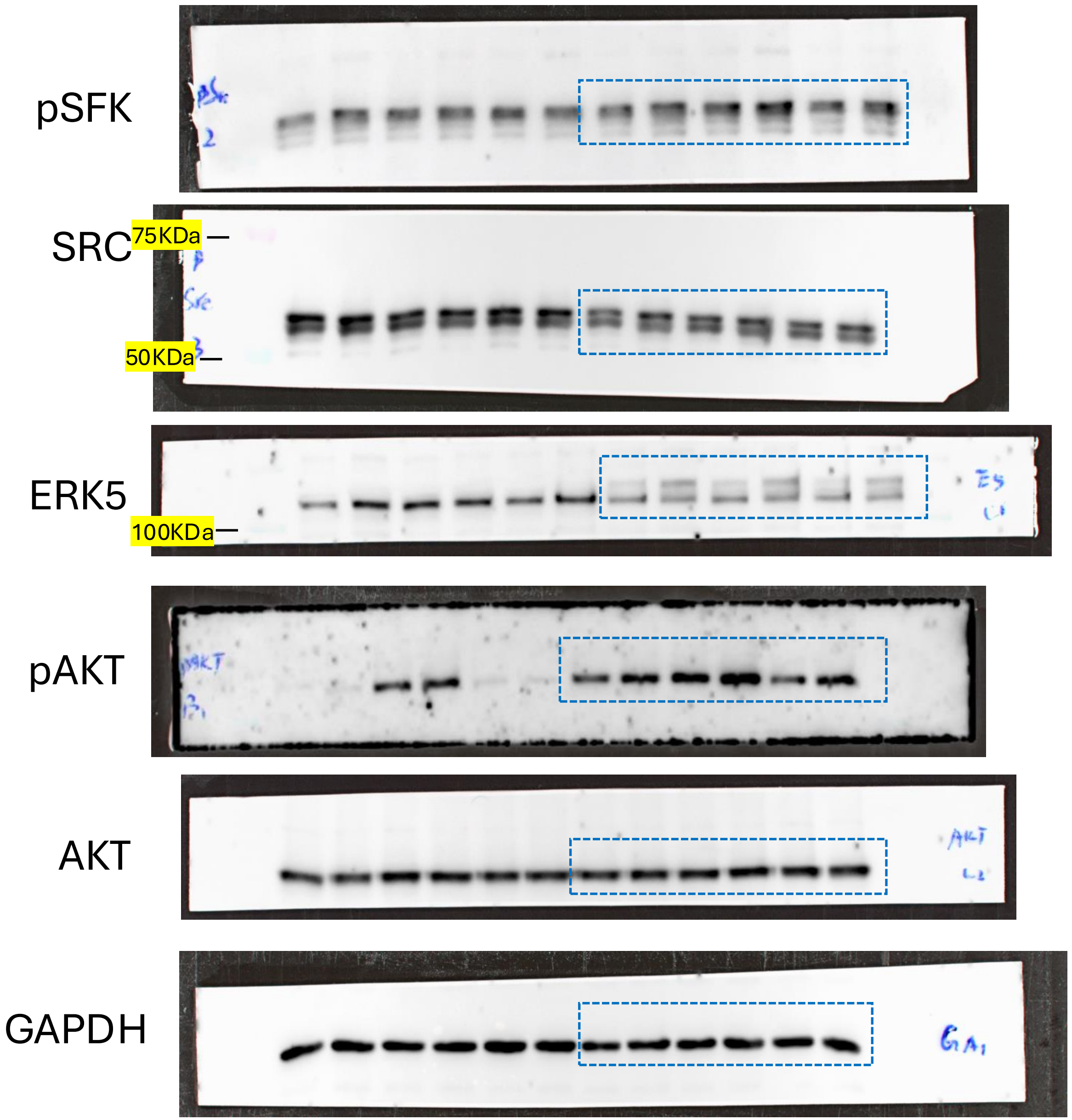

Full unedited blot/gel for Figure 7I

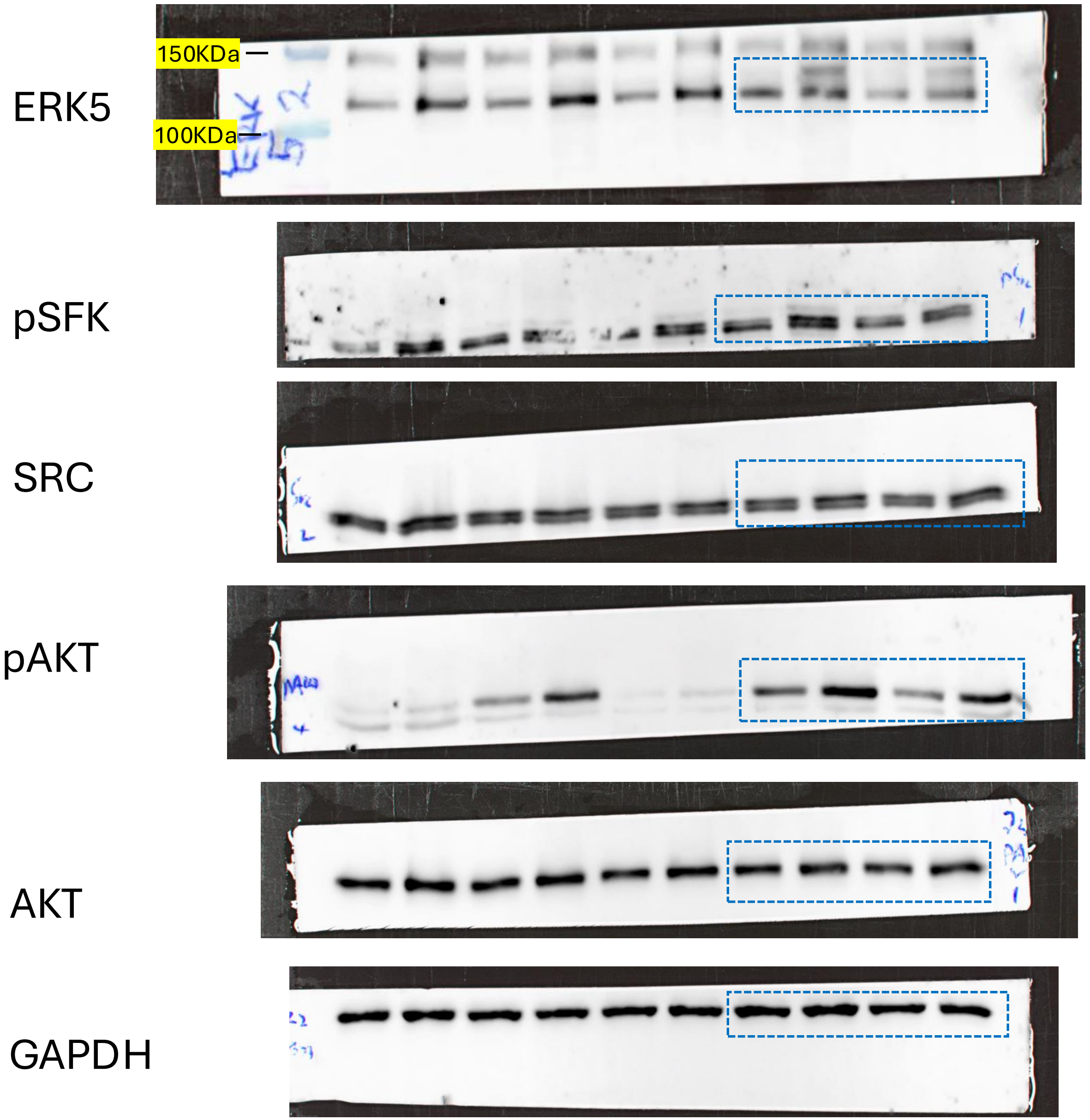

Full unedited blot/gel for Suppl Figure 1

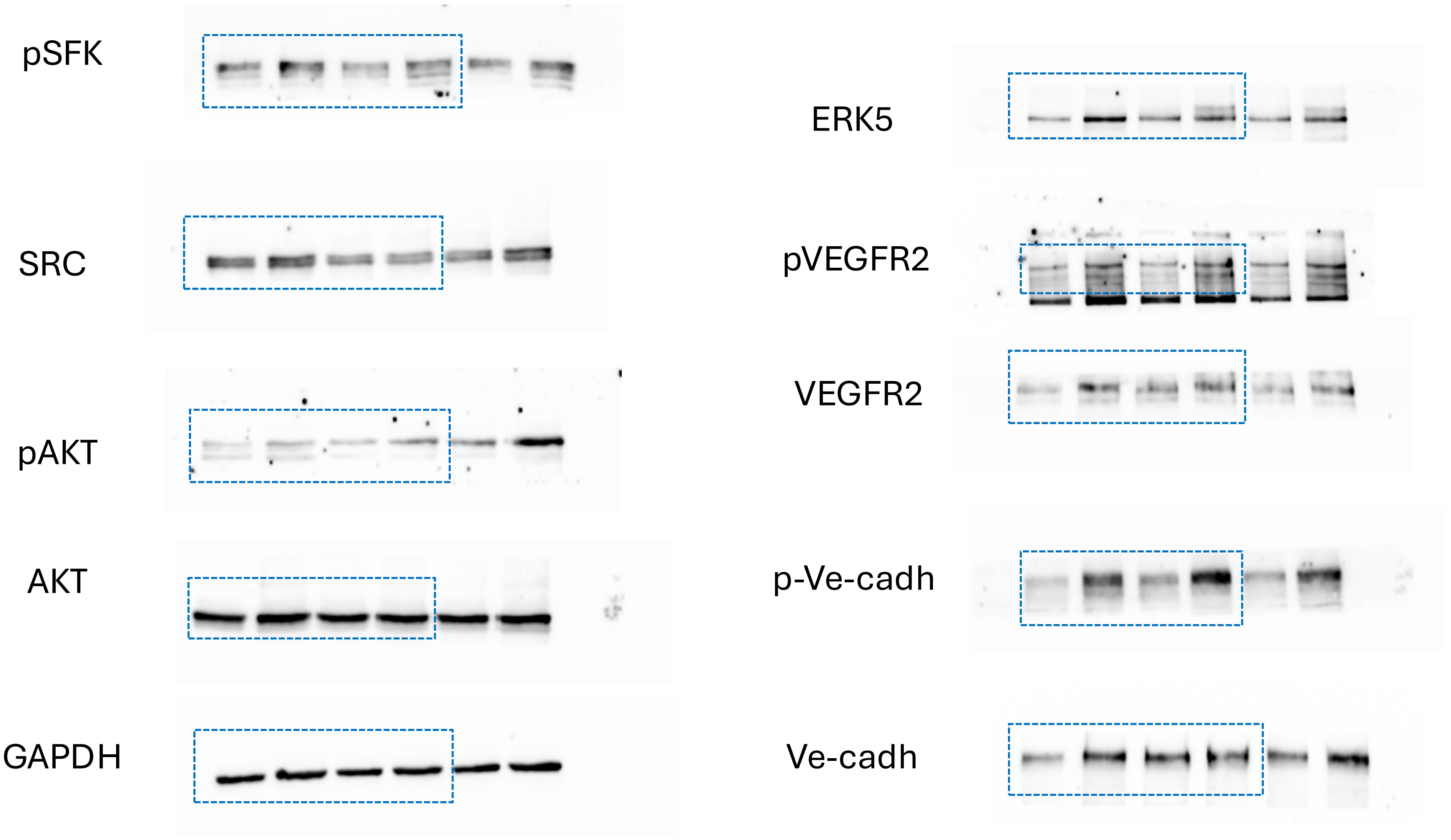

Full unedited blot/gel for Suppl Figure 2 A

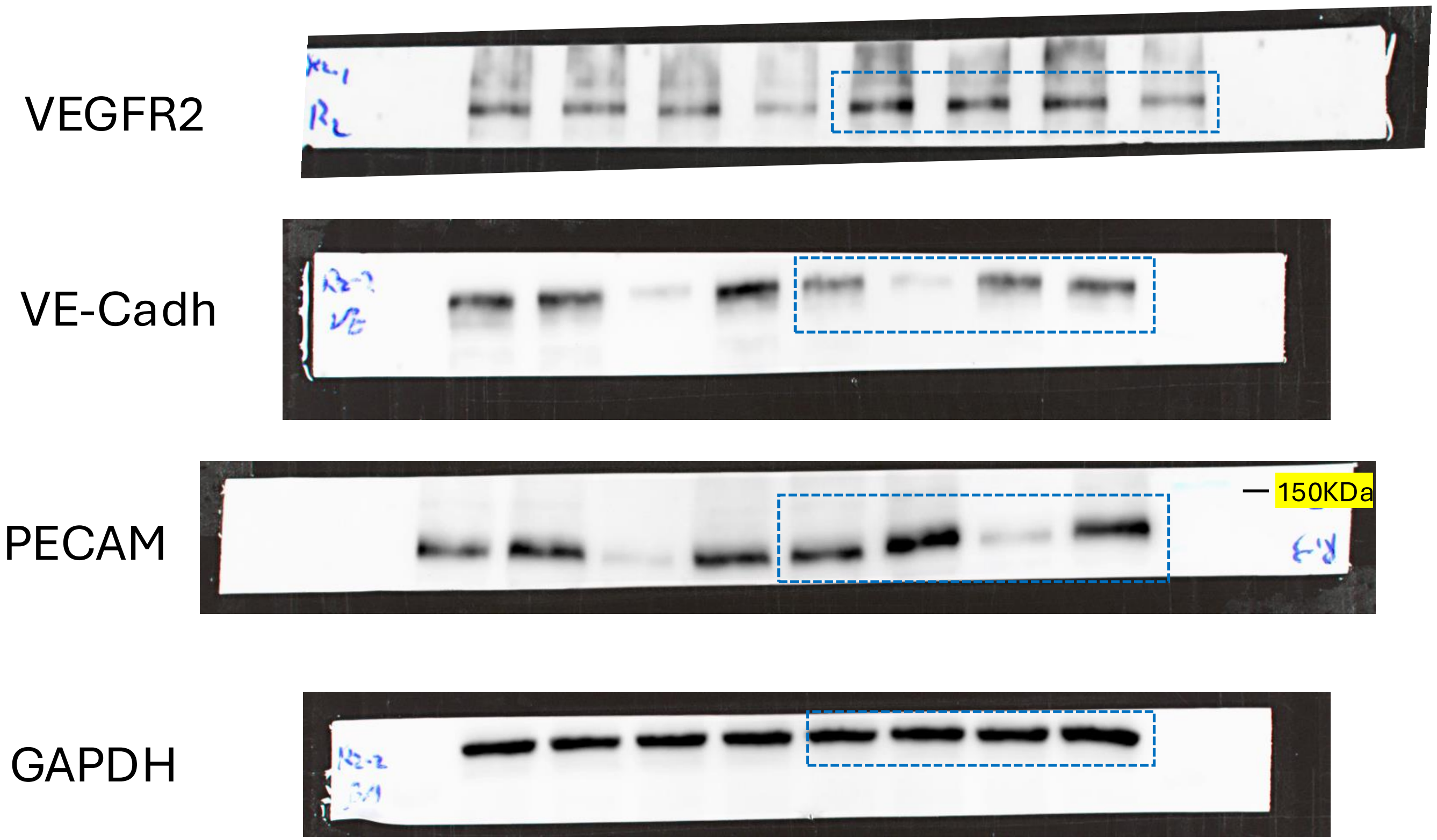

Full unedited blot/gel for Suppl Figure 2D

ERK5

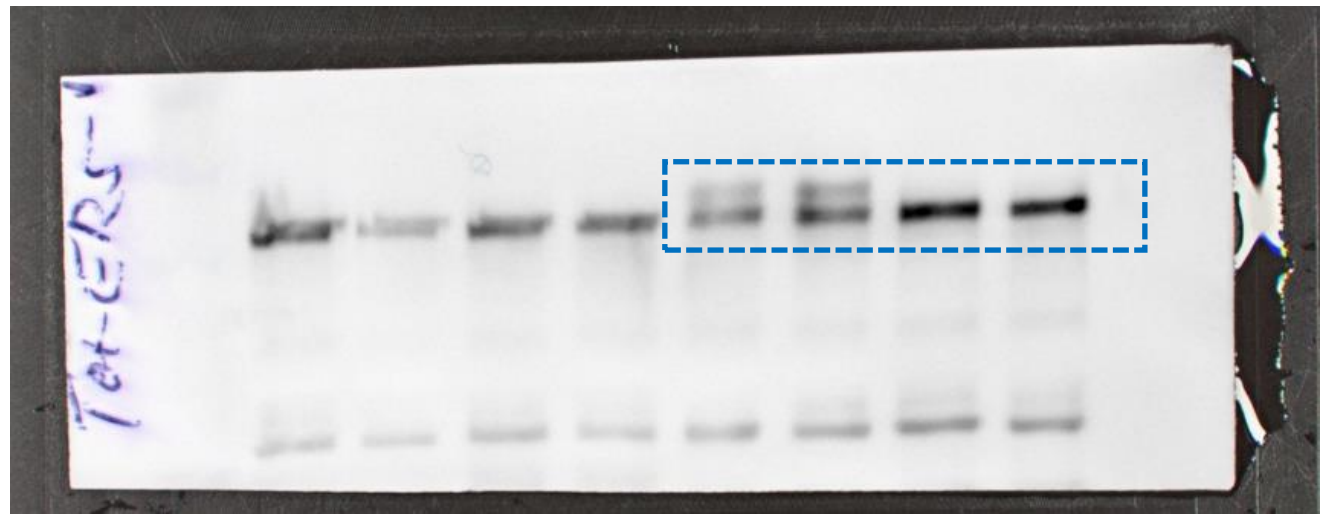

pAKT

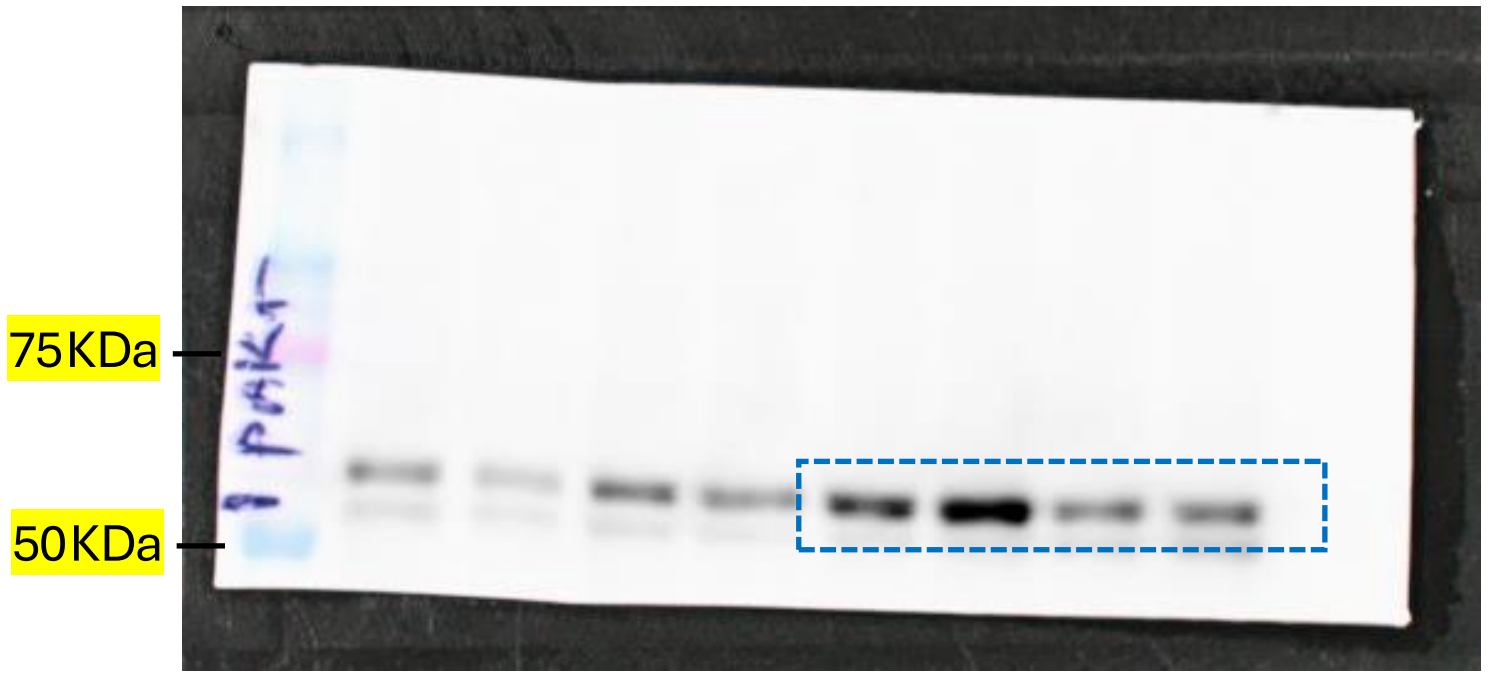

AKT

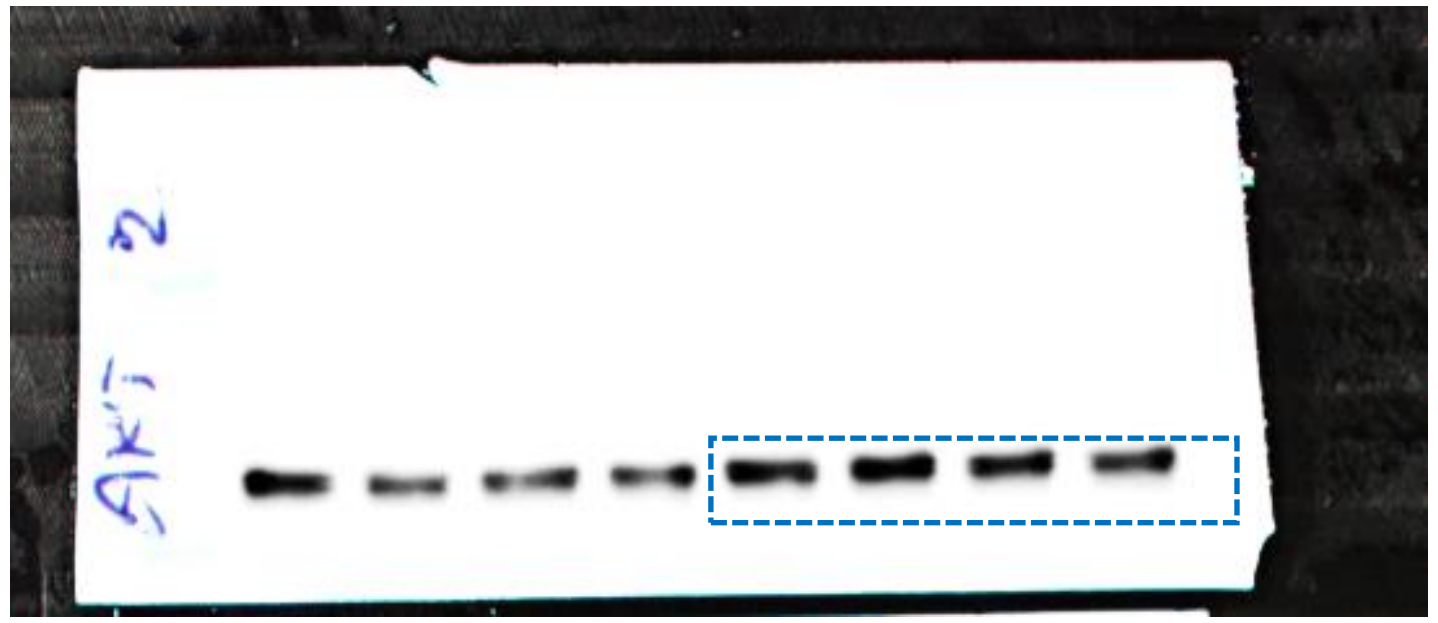

GAPDH

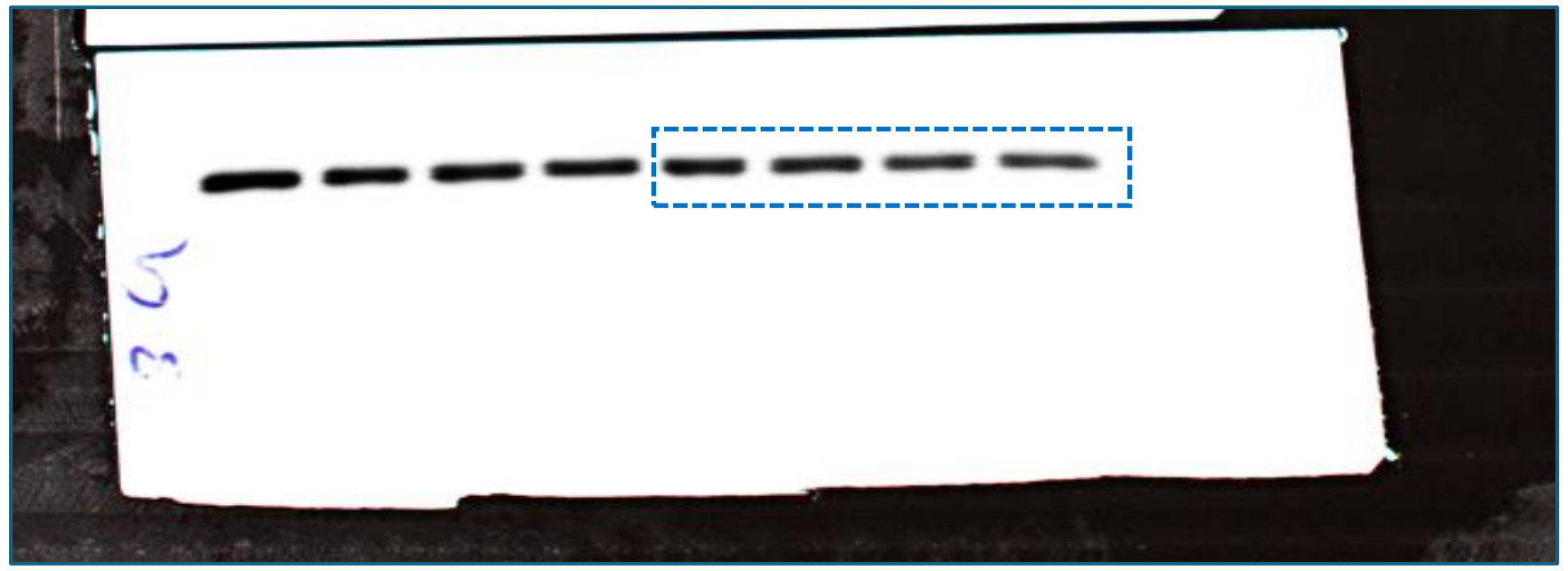

Full unedited blot/gel for Suppl Figure 3G

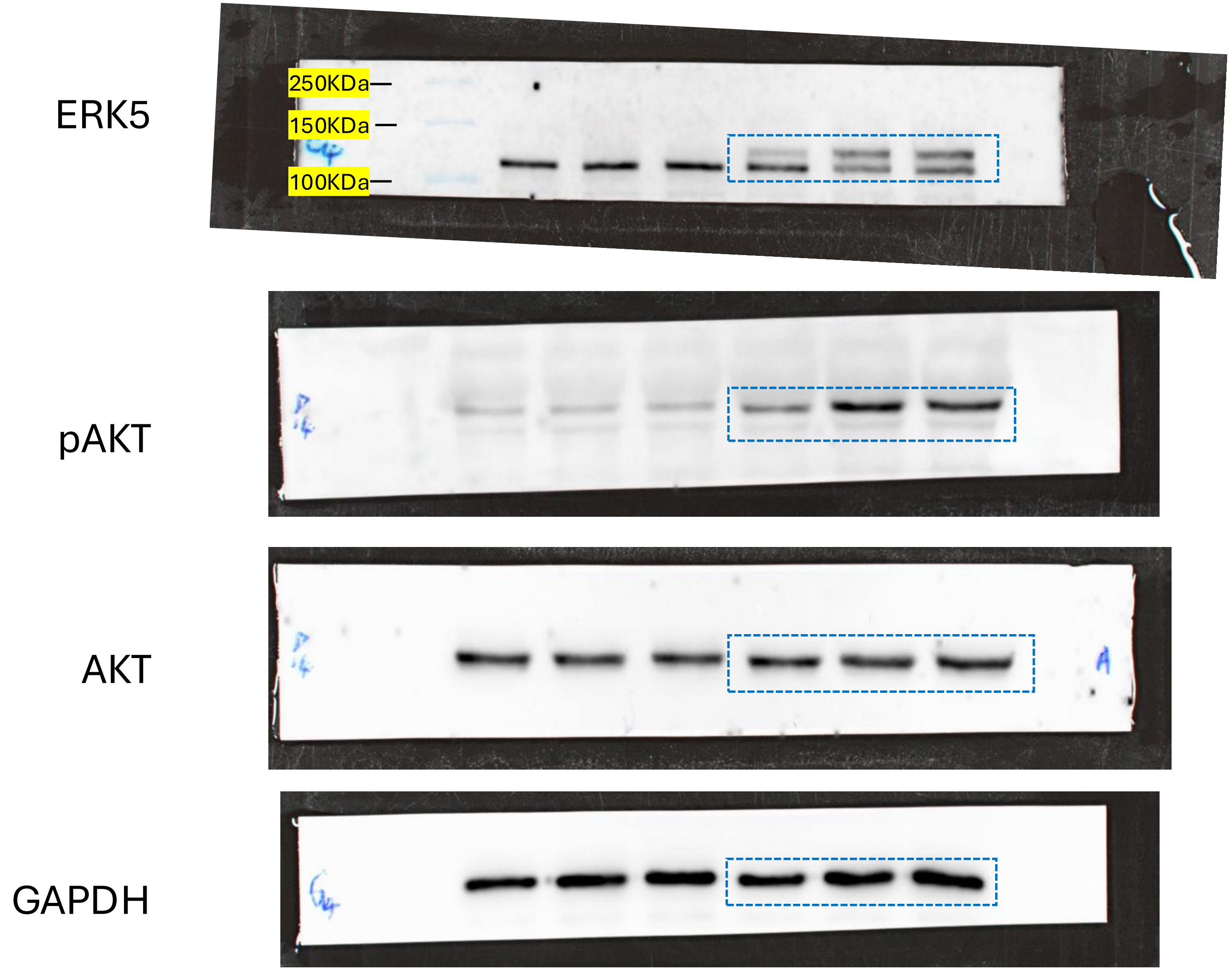

Full unedited blot/gel for Suppl Figure 4A

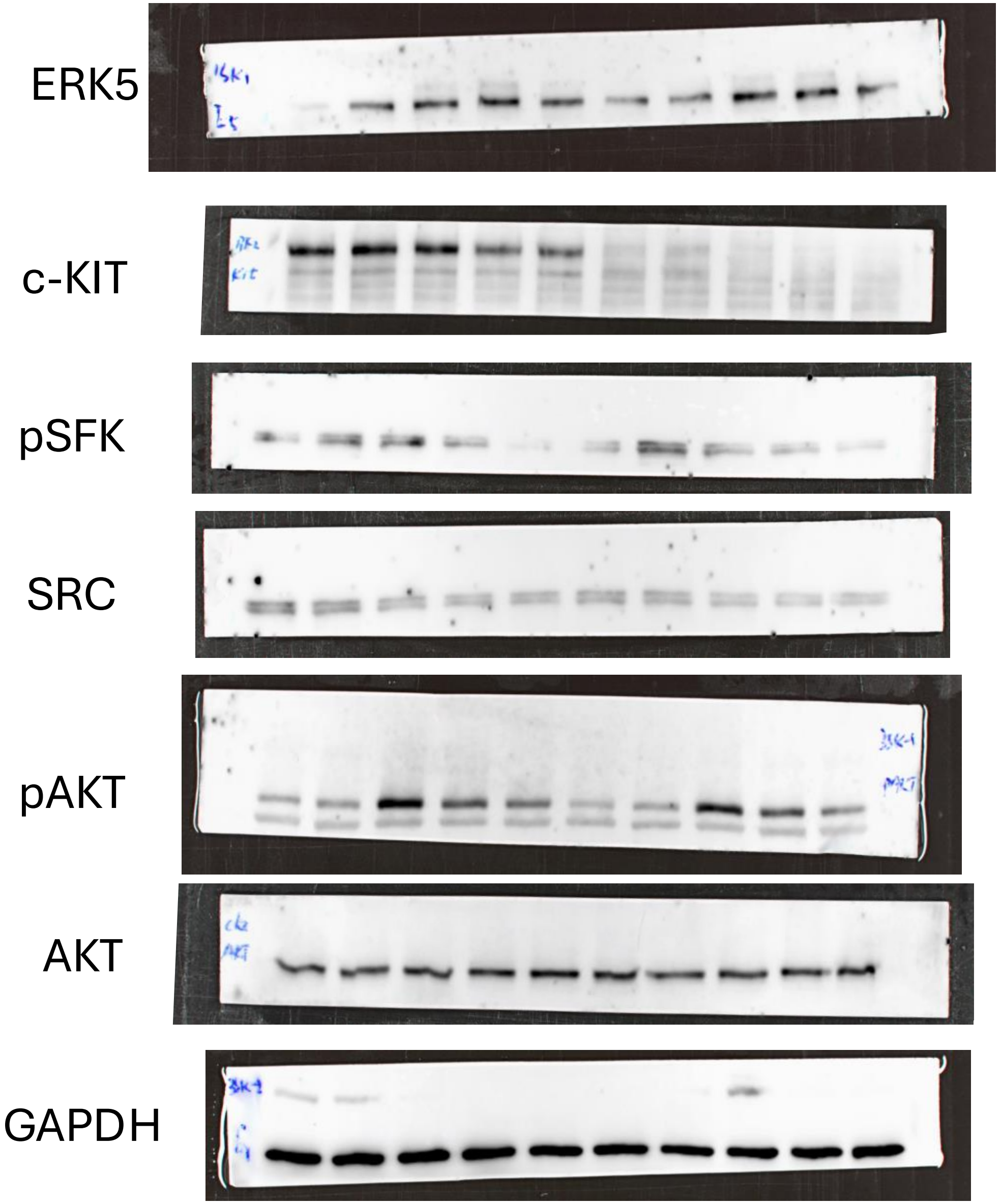

Full unedited blot/gel for Figure 5D

Control IgG IP

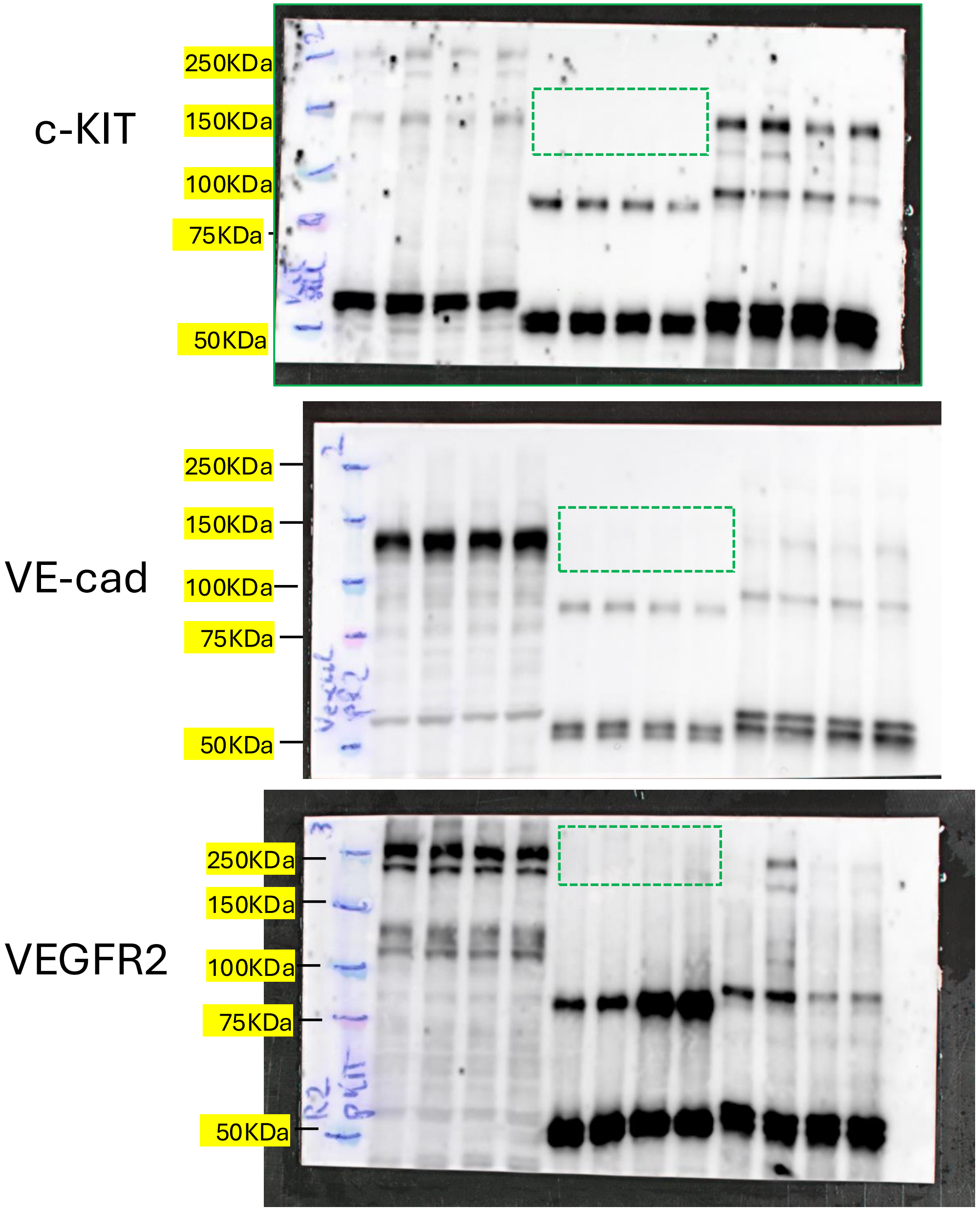

Full unedited blot/gel for Suppl Figure 4E

Control IgG IP

VE-cad

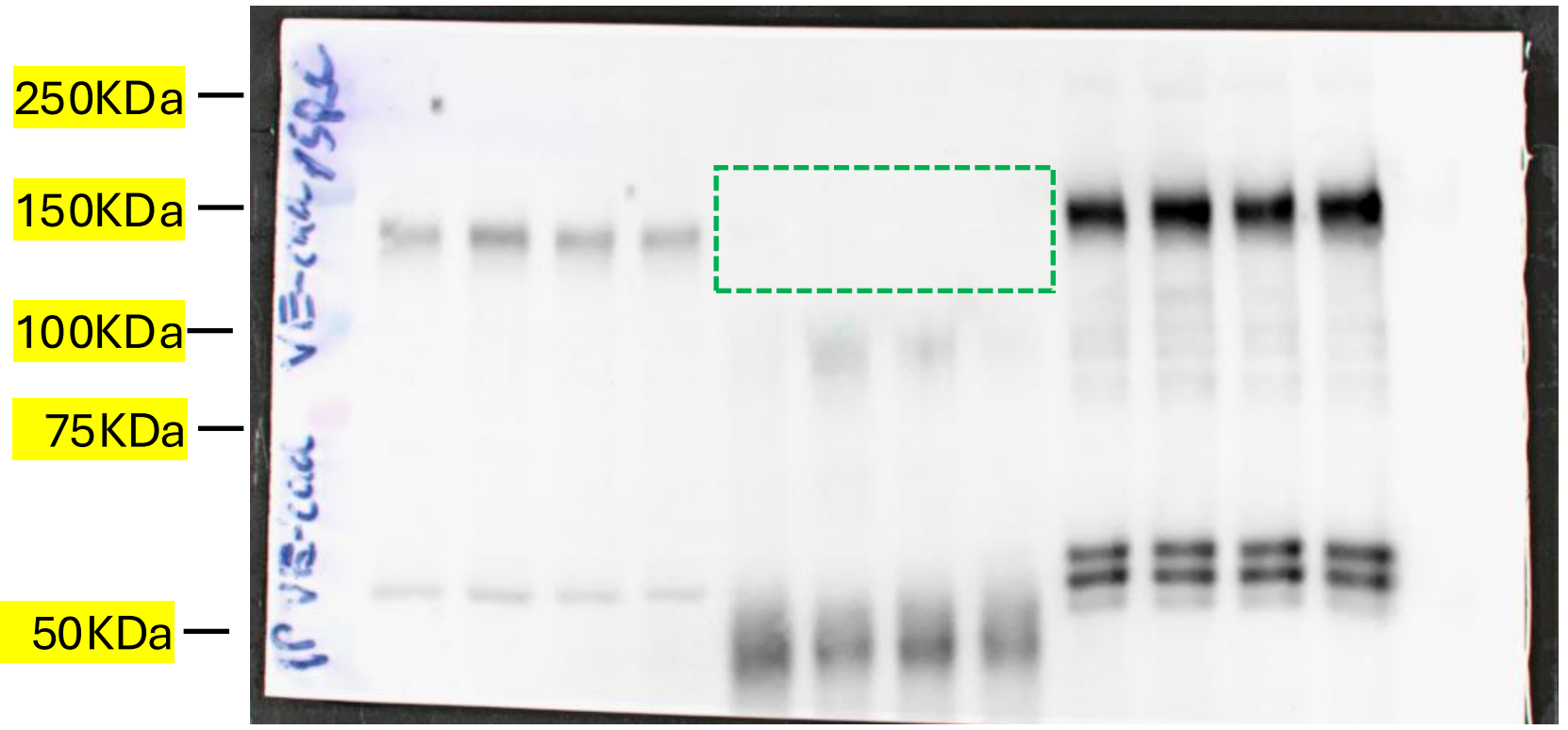

pTyr

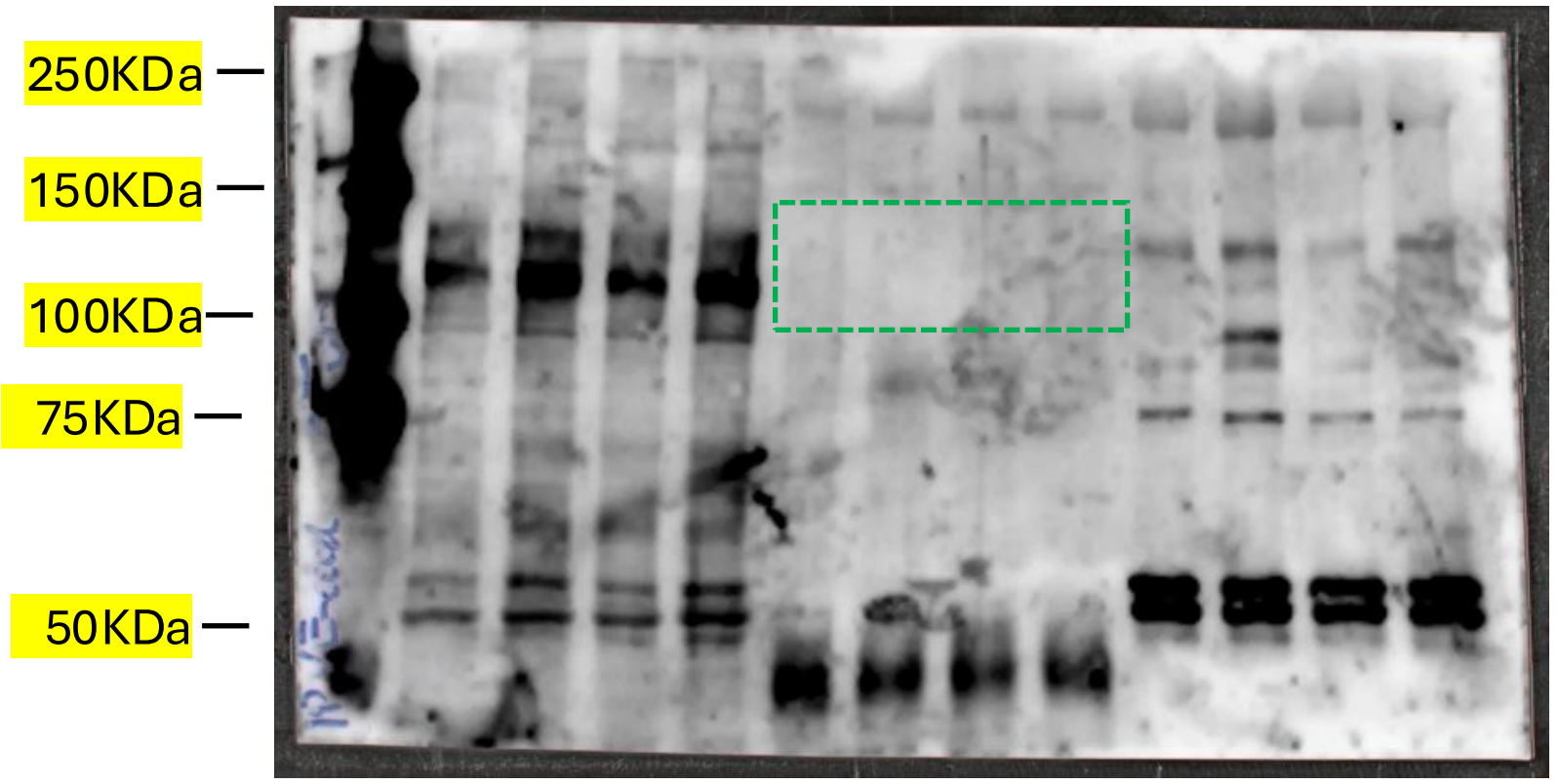

c-KIT

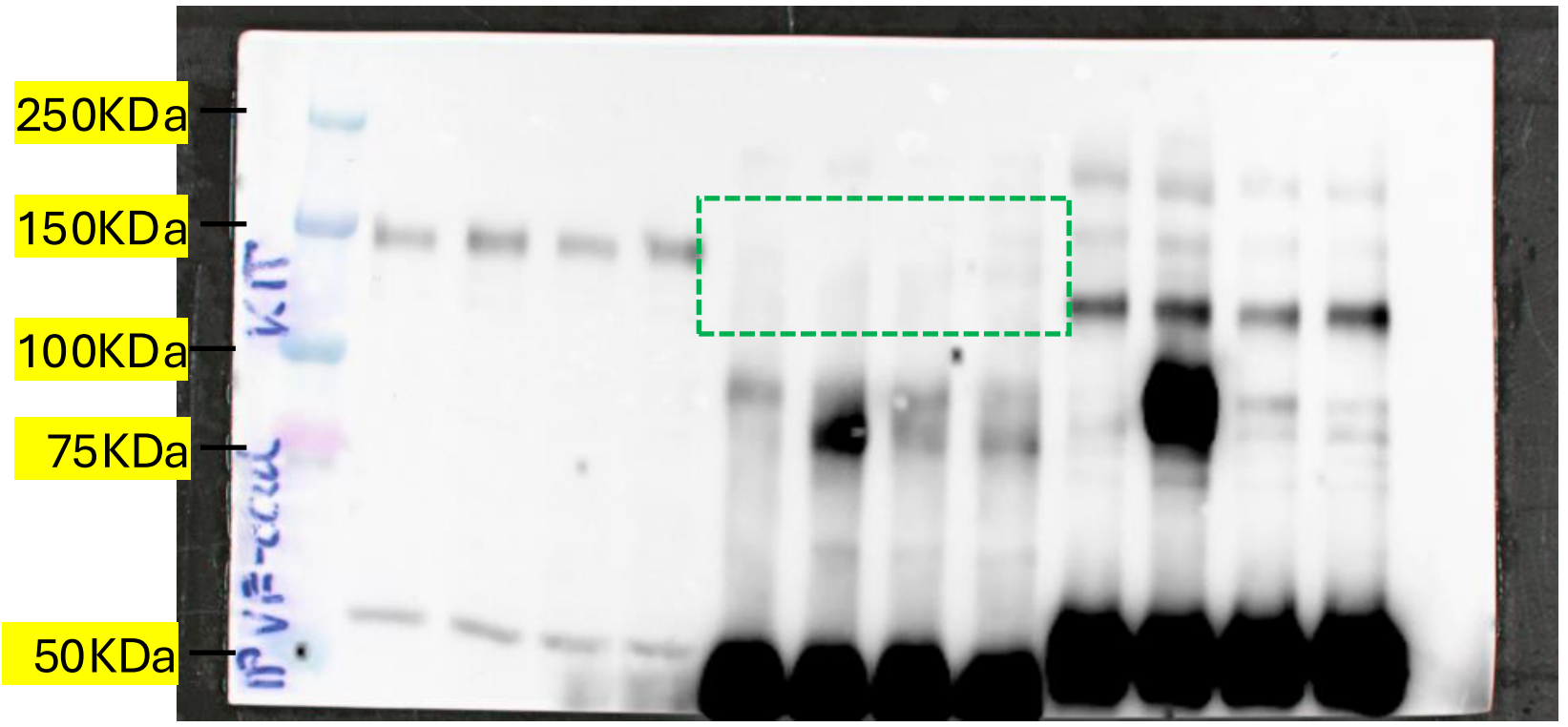

VEGFR2

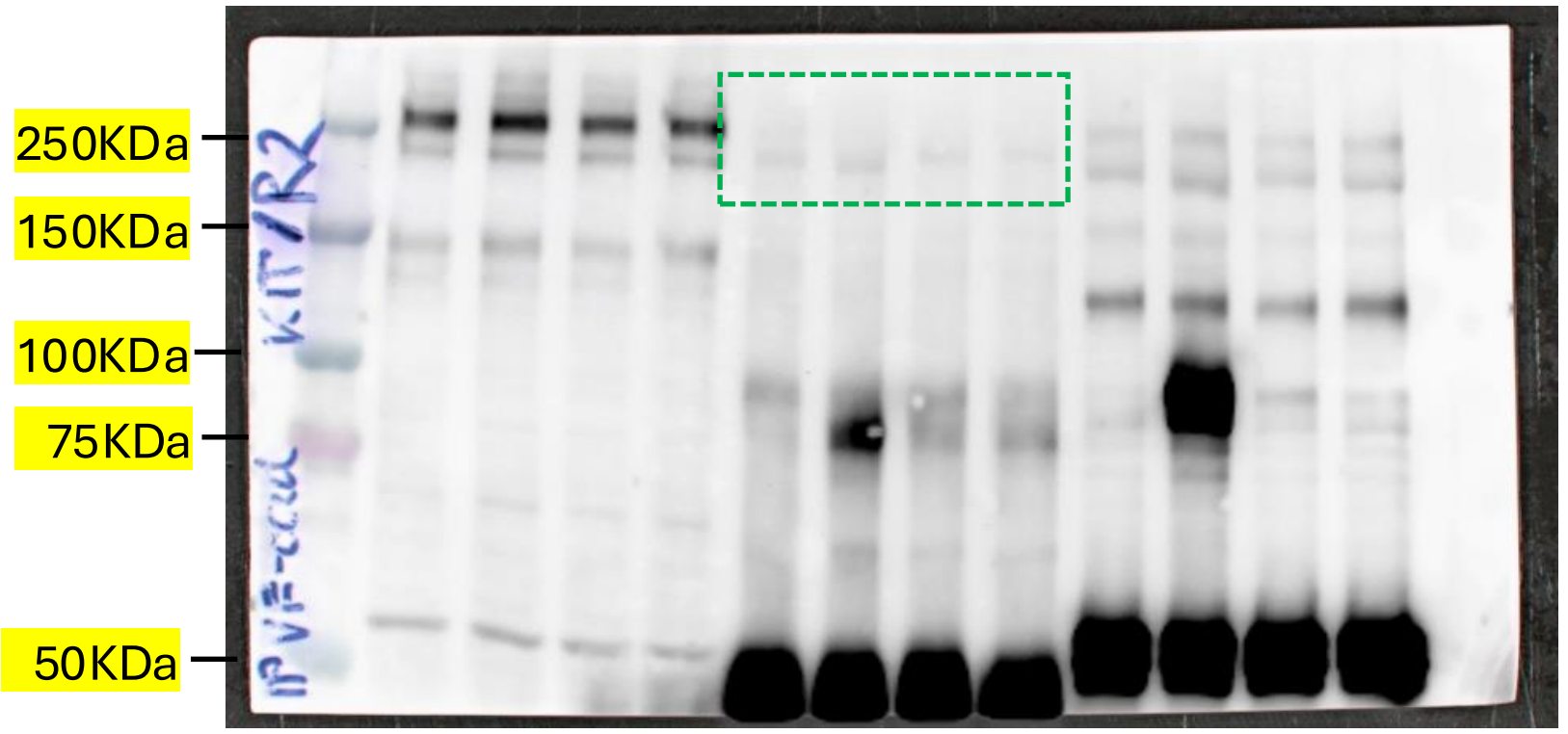

Full unedited blot/gel for Suppl Figure 5

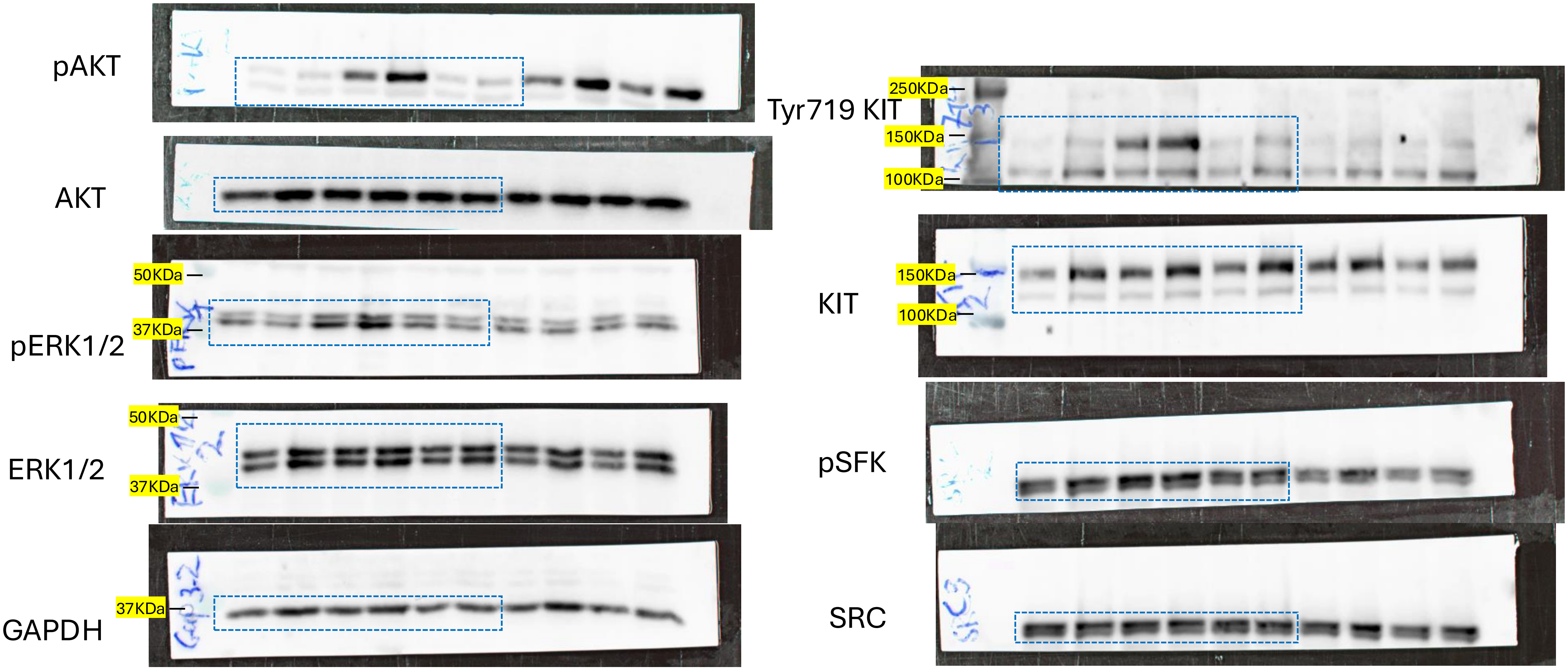

Supplement: Supplementary file 2 [file jci-136-197609-s348.pdf]
